# Differential HIF2α Protein Expression in Human Carotid Body and Adrenal Medulla under Physiologic and Tumorigenic Conditions

**DOI:** 10.3390/cancers14122986

**Published:** 2022-06-17

**Authors:** Lucía Celada, Tamara Cubiella, Jaime San-Juan-Guardado, Andrés San José Martínez, Nuria Valdés, Paula Jiménez-Fonseca, Ignacio Díaz, Jose María Enguita, Aurora Astudillo, Enol Álvarez-González, Luisa María Sierra, María-Dolores Chiara

**Affiliations:** 1 Institute of Sanitary Research of the Principality of Asturias, 33011 Oviedo, Spain; luciacelada@ispasturias.es (L.C.); tamaracubiella@gmail.com (T.C.); uo265392@uniovi.es (J.S.-J.-G.); andres.sanjosem@gmail.com (A.S.J.M.); nvaldes@fis.hca.es (N.V.); palucaji@hotmail.com (P.J.-F.); astudillo@hca.es (A.A.); enolalvglez@hotmail.com (E.Á.-G.); lmsierra@uniovi.es (L.M.S.); 2 CIBERONC (Network of Biomedical Research in Cancer), 28029 Madrid, Spain; 3 Department of Internal Medicine, Section of Endocrinology and Nutrition, Cabueñes University Hospital, 33394 Gijón, Spain; 4 Department of Medical Oncology, Central University Hospital of Asturias, 33011 Oviedo, Spain; 5 Department of Electrical Engineering, University of Oviedo, 33203 Gijón, Spain; idiaz@uniovi.es (I.D.); jmenguita@uniovi.es (J.M.E.); 6 Department of Functional Biology, University of Oviedo, 33006 Oviedo, Spain; 7 Institute of Oncology of the Principality of Asturias, University of Oviedo, 33006 Oviedo, Spain

**Keywords:** paraganglioma, pheochromocytoma, carotid body, adrenal medulla, hypoxia inducible factor, Von Hippel Lindau, succinate dehydrogenase

## Abstract

**Simple Summary:**

Thoraco-abdominal paraganglioma and pheochromocytoma (PPGL) are pathogenically linked to mutations in *SDH* genes. Loss-of-function of SDH has been associated with stabilization of HIF2α, which otherwise would be degraded via oxygen-sensitive mechanisms. SDH dysfunction also predispose to the development of paragangliomas arising at the carotid body or other head and neck paraganglia (HNPGL). Although PPGL and HNPGL share similar features, they have certain clinical and genetic peculiarities. By comparison of HIF2α expression in HNPGL and PPGL, we found that functional HIF2α is overexpressed in 80% of PPGLs, including those with *SDH* mutations as compared with non-tumor tissue. However, HIF2α is already highly expressed in the carotid body under physiologic conditions, and it is not overexpressed in HNPGL. These data suggest that selective HIF2α inhibitors already in clinical trials may benefit a wide spectrum of PPGL.

**Abstract:**

Hypoxia-inducible factors (HIF) 2α and 1α are the major oxygen-sensing molecules in eukaryotic cells. HIF2α has been pathogenically linked to paraganglioma and pheochromocytoma (PPGL) arising in sympathetic paraganglia or the adrenal medulla (AM), respectively. However, its involvement in the pathogenesis of paraganglioma arising in the carotid body (CB) or other parasympathetic ganglia in the head and neck (HNPGL) remains to be defined. Here, we retrospectively analyzed HIF2α by immunohistochemistry in 62 PPGL/HNPGL and human CB and AM, and comprehensively evaluated the HIF-related transcriptome of 202 published PPGL/HNPGL. We report that HIF2α is barely detected in the AM, but accumulates at high levels in PPGL, mostly (but not exclusively) in those with loss-of-function mutations in *VHL* and genes encoding components of the succinate dehydrogenase (SDH) complex. This is associated with upregulation of *EPAS1* and the HIF2α-regulated genes *COX4I2* and *ADORA2A*. In contrast, HIF2α and HIF2α-regulated genes are highly expressed in CB and HNPGL, irrespective of VHL and SDH dysfunctions. We also found that HIF2α and HIF1α protein expressions are not correlated in PPGL nor HNPGL. In addition, HIF1α-target genes are almost exclusively overexpressed in *VHL*-mutated HNPGL/PPGL. Collectively, the data suggest that involvement of HIF2α in the physiology and tumor pathology of human paraganglia is organ-of-origin-dependent and HIF1α-independent.

## 1. Introduction

Biochemical pathways involved in the cellular response to hypoxia have key roles on cancer development and metastasis [1]. The major hub where the oxygen-sensing pathways converge is represented by the hypoxia inducible factor, HIF, composed by any of the HIFα subunits (HIF1α, HIF2α, HIF3α) and HIFβ. HIF1α and HIF2α, encoded by *HIF1A* and *EPAS1* genes, respectively, are the most widely studied HIFα subunits. These proteins are degraded in the proteasome under normoxic conditions by a mechanism requiring active prolyl hydroxylase (PHD) enzymes and subsequent HIFα interaction with the Von Hippel Lindau (VHL) protein, a component of the protein complex with ubiquitin ligase E3 activity [2]. Hypoxia represses PHD activities leading to the stabilization and functional activation of the HIF complex. This occurs in cancer tissues when the tumor cells’ proliferation expands beyond the capacity of the tumor to increase new blood vessel formation.

A large number of reports have shown that hypoxia and HIF1α accumulation in the growing tumors have a negative impact on patient prognosis and response to therapies because of the transcriptional activation of genes involved in angiogenesis, proliferation, metabolism reprogramming, invasion and metastasis [3]. More recent discoveries have also emphasized that HIFα subunits may have a role in tumor initiation. Specifically, germline genetic defects affecting the hypoxia signaling pathways predispose to the development of certain neoplasia, mainly clear cell renal cell carcinomas (ccRCC), paragangliomas and pheochromocytomas, via mechanisms likely involving HIFα factors [4,5].

Paragangliomas and pheochromocytomas are rare tumors arising at neural-crest-derived tissues of parasympathetic or sympathetic origin, such as the carotid body (CB) and the adrenal medulla (AM), which are the prototypical oxygen and stress organ sensors of mammals. Parasympathetic paragangliomas include tumors developed at the CB or other paraganglia of the head and neck region (hereafter referred to as HNPGL). Sympathetic paraganglioma develop in the paraganglia of the sympathetic nervous system at the thorax or abdomen; tumors arising at the AM are known as pheochromocytoma, and together with sympathetic thoraco-abdominal paraganglioma, are referred to here as PPGL. At the physiological level, studies in mice models have shown that HIF2α has prominent functions in the development of the CB and its responsiveness to acute hypoxia [6,7,8]. It is also essential for the growth, differentiation, and function of the sympathoadrenal lineage [9,10,11,12]. However, the roles of HIF1α and HIF2α in HNPGL and PPGL development have been more poorly defined.

PPGL are hereditary in about 40% of cases affecting, among other genes, activators of the oxygen-sensing pathways, mainly *VHL* and genes encoding for components of the succinate dehydrogenase (SDH) complex (*SDHB*, *SDHD*, *SDHC*, *SDHA* and *SDHAF2* abbreviated hereafter as *SDH*) [13]. In addition, gain-of-function mutations in *EPAS1* have been recently identified [14,15,16,17,18]. The activation of the HIF-signaling pathway has been reported to occur in PPGL with *SDH*, *VHL* or *EPAS1* mutations, a condition that has been termed pseudohypoxia, given that it occurs in highly vascularized, non-hypoxic tumors. These findings inspired the hypothesis that HIFα subunits have an oncogenic role in PPGL and HNPGL. However, there are still many uncertainties that hamper understanding of the physiopathological significance of pseudohypoxia in PPGL and HNPGL, as well as hindering the translation of this knowledge into clinical benefit for patients. For instance, most previously reported studies have focused on PPGL, whereas the association of pseudohypoxia and *SDH*-mutations in HNPGL has been mostly unexplored. Although PPGL and HNPGL share similar features, they have certain clinical and genetic peculiarities. For instance, metastasis and secretory tumors are more frequently found in patients with PPGL than HNPGL [19]. At the genetic level, mutations in *VHL* are frequently associated with PPGL, but they are extremely rare in HNPGL, which are more frequently associated with *SDH* mutations [20]. Moreover, gain-of-function mutations in *EPAS1* identified in PPGL have not been found thus far in HNPGL. Therefore, given the heterogeneities associated with tumor genotype and lineage of origin, it is still uncertain whether this is reflected in a differential expression of HIFα subunits and their target genes. There are several indicators suggesting that this may be the case. For instance, many of the canonical hypoxia-related genes were not found to be overexpressed in HNPGL, suggesting that the HIF-related genetic module has certain specific features in parasympathetic tumors versus sympathetic tumors [21]. Furthermore, the association of *SDH* mutations with activation of HIF1α or HIF2α, or of both transcription factors together, has been a subject of debate with conflicting published data in cell lines and tumor tissues [22,23,24]. The fact that these two transcription factors have specific and unique targets in different cell types may partially explain the discrepancies, and certainly adds complexity to the field. Therefore, a comprehensive knowledge of which hypoxia/HIF-related genes, among the entire constellation identified so far, are the ones that are overexpressed in pseudohypoxic PPGL and HNPGL, could help fill in those gaps. Furthermore, unraveling whether it is HIF1α or HIF2α that is activated in pseudohypoxic HNPGL and PPGL, or whether it is both proteins together, could also add scientific knowledge relevant to the therapeutic management of patients. In this sense, several recently developed pharmacological strategies specifically targeting any of the two HIFα subunits are providing promising results in syndromes caused by *VHL* or *EPAS1* mutations [25,26].

In this report, we aimed to define and compare the pseudohypoxic profile of PPGL and HNPGL by relating them to their genotypic origin, cancer-associated hypoxic phenotype and HIF2α expression. We performed in silico analysis of publicly available transcriptomic data, and then used this resource to identify transcriptional features of PPGL and HNPGL to correlate phenotypic diversity with genotypic heterogeneity and organ-of-origin.

## 2. Materials and Methods

### 2.1. Tumor Samples and Cell Line

Surgically resected specimens of formalin-fixed and paraffin-embedded (FFPE) tissues were retrospectively obtained from 50 patients with PPGL/HNPGL who were diagnosed and treated between 2009 and 2020 at the Central University Hospital of Asturias (Oviedo, Spain). Tumor samples included 55 primary tumors and 7 metastases (Supplementary Table S1). Informed consent was obtained from each patient. The study was approved by the Ethical Committee of the Central University Hospital of Asturias. The methods were carried out in accordance with the approved guidelines and the principles expressed in the Declaration of Helsinki. Clinical data were collected from the patients’ medical records. The gene mutation data were retrieved from our previous study [27]. Sequencing of tumor DNA was performed with an in-house-developed targeted sequencing panel, including all known PPGL-susceptibility genes. Non-tumoral carotid bodies had been obtained from organ donors, as described previously [21]. Non-tumoral adrenal medulla from organ donors were newly obtained for the current study.

PC12 cells were grown in Dulbecco’s modified Eagle’s medium supplemented with 15% fetal bovine serum, 100 units/mL penicillin and 100 μg/mL streptomycin. Cells were exposed to 1 mM water-soluble mono-methyl hydrogen succinate (Sigma-Aldrich, St. Louis, MO, USA) for different times (1–48 h). Where indicated, cells were pretreated with 1 mM mono-methyl hydrogen succinate for 12 h. Subsequently, 5 µM PT2385 (Selleck Chemicals, Munich, Germany) was added and cells were incubated for additional 36 h. See Appendix A for details on the SCC38 cell line culture conditions.

### 2.2. The Cancer Genome Atlas Data Analysis and Gene Expression Data. PPGL

RNA-seq data were retrieved from The Cancer Genome Atlas (TCGA) data portal (https://portal.gdc.cancer.gov/projects (accessed on 15 February 2020)). For HNPGL, we used published microarray data obtained with the Affymetrix GeneChip Human Genome U133 Plus 2.0 Arrays [21]. See [28,29,30,31] and Appendix A for details on gene expression analysis.

### 2.3. Immunohistochemistry and Immunofluorescence

FFPE human tissues from surgically treated patients were cut into 4 μm sections and mounted on poly-L-lysine coated slides. Antigen retrieval of deparaffinized tissues was performed by heating at 95 °C for 20 min in a Dako PT link platform (Dako Denmark A/S, Glostrup, Denmark) with EnVision™ FLEX target retrieval solution pH 9 for HIF1α, HIF2α and S100 immunostainings or pH 6 for TH immunohistochemistry. Stainings were carried out automatically according to the manufacturer’s instructions, using a Dako Autostainer Plus and the Dako EnVision™ Flex detection systems (Dako Denmark A/S). Tissue slides were incubated with the following primary antibodies: rabbit IgG anti-HIF2α antibody (ab199, lot number: GR3374543-1, Abcam, Cambridge, UK) for 30 min at 1:50 dilution; mouse IgG anti-HIF1α antibody (BD610958 clone 54, lot number: 1011250BD, BD Biosciences, San Jose, CA, USA) for 60 min at 1:50 dilution plus EnVision™ Flex+ Mouse LINKER (Dako Denmark A/S) for 15 min; rabbit anti-S100 antibody (IS504, Ready-to-Use, Dako Denmark A/S) for 20 min and rabbit anti-TH antibody (ab6211, Abcam) for 30 min at 1:80 dilution. Slides were counterstained with hematoxylin. Positive and negative controls were also included. Prior to immunohistochemical analysis in tumor tissues, the specificity of the HIF2α antibody was confirmed by using approaches described in Supplementary Figure S1. For HIF1α immunohistochemistry, quantification was performed as previously described [32]. Images were analyzed randomly by 3 of the authors (L.C., A.A., and M.-D.C.) without knowledge of clinicopathological data. For immunofluorescence, cells were fixed in 4% paraformaldehyde for 15 min and permeabilized with PBS containing 0.1% Triton X-100. Rabbit IgG anti- HIF2α was used at 1:50 dilution. Anti-rabbit IgG Alexa Fluor 555 was used as secondary antibody at 1:500 dilution. Images were taken using a Zeiss AxioObserver Z1 microscope (Carl Zeiss, Oberkochen, Germany).

### 2.4. Western Blot Analyses 

Protein extracts were obtained from cultured cells at 80%–90% confluence. Membranes were probed with rabbit anti-HIF2α (Abcam) at 1:250 dilution, rabbit anti-HIF1α (Novus Biologicals, Minneapolis, MN, USA) at 1:500 dilution or mouse anti-β-actin (Sigma-Aldrich) at 1:10,000 dilution. Bound antibodies were detected by using IRDye 800 CW or IRDye 700 IgG secondary antibodies (LI-COR Bioscience, Lincoln, NE, USA) at 1:10,000 dilution. Odyssey Fc Imaging System (LI-COR Bioscience) was used for image acquisition and densitometry analysis.

### 2.5. Quantitative Real-Time RT–PCR

RNA isolation was performed with a mirVana RNA isolation kit (Invitrogen, Thermo Fisher Scientific, Madrid, Spain). cDNA was synthesized using Maxima First Strand cDNA synthesis kit (Thermo Fisher Scientific). Quantitative PCR was performed using TaqMan probes for *COX412*, *NDUFA4L2*, *ADORA2A, SLC2A1* and *ENO1* (Thermo Fisher Scientific). Relative expression to peptidylprolyl isomerase A (*PPIA*) target gene was assessed to normalize RNA input amounts and perform relative quantifications.

### 2.6. Cell Viability Assays

Cells were seeded in a 96-well plate (10,000 cells/well) before exposure to 1 mM mono-methyl hydrogen succinate for 12 h. After pre-treatments, cells were incubated with 5 μM of PT2385 for 36 h. Cell viability was analyzed by using the CellTiter 96^®^ AQueous One Solution Cell Proliferation Assay (Promega, Madison, WI, USA).

## 3. Results

### 3.1. Defining the HIF-Related Transcriptome of Pseudohypoxic PPGL

Most of our knowledge on the HIF-related transcriptome in cancer has been settled, thanks to normal-cell and cancer-cell-based analyses, as well as studies on animal models and cancer tissues. Despite this progress, there is not a unified hypoxia-related gene signature in cancer that can be used to explore and define the gene expression module specific to pseudohypoxia. Moreover, the number of genes related to hypoxia is continuously expanding, and the regulation of many HIF targets is tissue-type specific. Therefore, to obtain a precise definition of the pseudohypoxic-related hallmark of PPGL, we decided to first construct a minimum and reliable hypoxia/HIF gene expression module in cancer that could be used to define the HIF-related transcriptome of pseudohypoxic PPGL. To this end, we took a pan-cancer perspective, resorting to the publicly available TCGA data, which includes over 10,000 individual tumors and 21 types of cancers.

**Figure 1:**
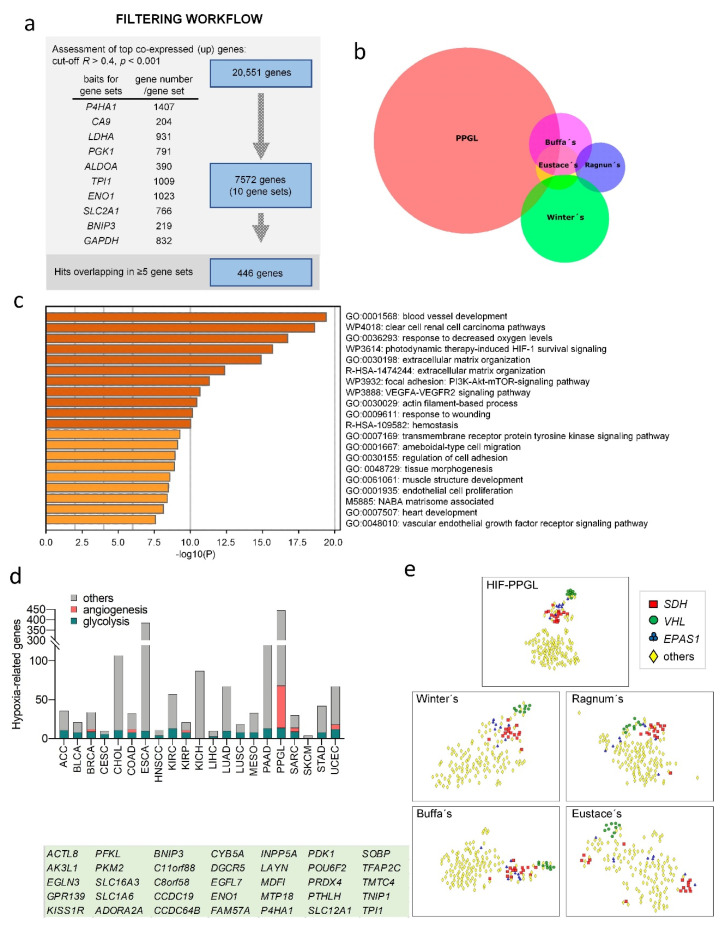
Defining the HIF-related transcriptome of paragangliomas. (**a**) Study design. Ten gene sets were obtained by correlation analysis of the indicated HIF-related genes and the whole RNA-seq data of PPGL included in the TCGA project (*R* > 0.4, *p* < 0.001). This analysis only considered upregulated genes (up). A total of 446 genes overlapped in ≥5 gene sets. (**b**) Venn diagram showing overlaps of the HIF-PPGL, Buffa’s, Winter’s, Eustace’s and Ragnun’s HIF-related signatures. (**c**) Top enriched Gene Ontology (GO) biological process and pathway terms of HIF-PPGL signature colored by p-values. (**d**) Number of hypoxia-related genes identified in the indicated cancer types included in the TCGA project (Illumina HiSeq pan-cancer normalized). The number of genes involved in glycolysis and angiogenesis are highlighted in green and red, respectively. (**e**) t-SNE plots of TCGA-PPGL based on the expression of the genes included in the HIF-PPGL, Buffa’s, Winter’s, Eustace’s and Ragnun’s signatures. The 35 genes used for HIF-PPGL tSNE analysis (genes overlapping in ≥9 of the gene sets indicated in panel (**a**)) are indicated. ACC, adrenocortical carcinoma; BLCA, bladder urothelial carcinoma; BRCA, breast invasive carcinoma; CESC, cervical squamous cell carcinoma and endocervical adenocarcinoma; CHOL, cholangiocarcinoma; COAD, colon adenocarcinoma; ESCA, esophageal carcinoma; HNSCC, head and neck squamous cell carcinoma; KIRC, kidney clear cell cancer cell carcinoma; KIRP, kidney renal papillary cell carcinoma; KICH, kidney chromophobe; LIHC, liver hepatocellular carcinoma; LUAD, lung adenocarcinoma; LUSC, lung squamous cell carcinoma; MESO, mesothelioma; PAAD, pancreatic adenocarcinoma; SARC, sarcoma; SKCM, skin cutaneous melanoma; STAD, stomach adenocarcinoma; UCEC, uterine corpus endometrial carcinoma.

Using this approach (see Appendix A for details), we identified the 10 well-known HIF-target genes (*P4HA1*, *LDHA*, *BNIP3*, *CA9*, *PGK1*, *SLC2A1*, *ENO1*, *ALDOA*, *TPI1* and *GAPDH*) that were the most consistently overexpressed genes in hypoxic cancers. These genes were selected and used as baits to construct a correlation matrix using the RNAseq data of the PPGL included in the TCGA database (see Appendix A for details and Supplementary Table S2). The top-ranking co-expressed genes (correlation coefficient *R* > 0.4; *p* < 0.001) overlapping in at least five of the ten gene sets were defined as the HIF associated profile of PPGL (HIF-PPGL, Figure 1A). This signature contained 446 genes, including known targets of HIF1α (*ALDOA*, *BNIP3*, *CA9*, *ENO1*, *HK2*, *LDHA*, *PFKL*, *PGM1*, *PKM*, *SLC2A1*) and HIF2α (*ADORA2A*, *CACNA1H*, *FLT1*) and genes known to be downregulated in paraganglia of *EPAS1* knockout mice (*NDUFA4L2*, *COX4I2*) (Supplementary Table S3) [8,33]. In comparison with previously reported cancer-associated hypoxia-related signatures (Buffa [34], Winter [35], Eustace [36] and Ragnum [37]), only 6% of the genes in the HIF-PPGL profile were also in any of those signatures (Figure 1B, Supplementary Table S4) and these were mostly involved in glycolysis and HIF1α pathway, indicating that genetically determined pseudohypoxia and cancer-associated genuine hypoxia have different features. Enrichment analysis of functional categories, analyzed using the Metascape^®^ bioinformatics tool, revealed that the most significant enrichment of the HIF-PPGL signature concerned genes involved in angiogenesis/vasculogenesis (Figure 1C and Supplementary Table S3). The HIF1α pathway and glycolysis were also among the enriched pathways, but with much less significance in comparison to the predominant glycolytic profile described in most hypoxic cancer types.

To compare the HIF-PPGL signature with the hypoxia-driven HIF signature identified in other types of tumors, we used a similar approach in 21 types of cancers included in the TCGA database. This yielded a HIF signature for each cancer type. Gene Ontology term enrichment analysis confirmed that the most significantly enriched biological process in all analyzed cancers was related to glycolysis (Figure 1D). Genes involved in angiogenesis were found enriched in some cancers, but to a much lesser extent than observed in PPGL. Therefore, HIFα subunits drive a differing balance of angiogenic and metabolic features in PPGL in comparison with most cancer types.

### 3.2. HIF2α-Related Gene Expression in Genetically Determined Pseudohypoxic PPGL

Burnichon et al. previously reported that PPGL with *VHL* or *SDH* mutations formed a separated cluster from tumors lacking these mutations. Nevertheless, they also showed that tumors with *VHL* mutations (*VHL*-PPGL) formed an independent cluster separated from *SDH*-mutated PPGL (*SDH*-PPGL) [38]. Despite these studies, improved understanding of the pseudohypoxia-phenotypic diversity of PPGL is still lacking [39,40]. In addition, it is not clear whether tumors with *EPAS1* mutations are more closely related to *VHL*-PPGL, *SDH*-PPGL, or both. Here, we performed unsupervised clustering analysis using the 35 top-ranking genes contained in the HIF-PPGL signature (genes overlapping in ≥9 gene sets) (Figure 1E). t-distributed stochastic neighbor embedding (t-SNE) plots were used for visualization. This analysis confirmed that PPGLs carrying *SDH*, *VHL* or *EPAS1* mutations were more closely related with each other than with non-pseudohypoxic tumors. We also found that pseudohypoxic PPGL split into two clusters, one containing *VHL*-PPGL and the other containing *SDH*-PPGL plus *EPAS1*-mutated PPGL (*EPAS1*-PPGL) (Figure 1E). tSNE analysis using Buffa’s, Winter’s, Eustace’s or Ragnum’s signatures did not provide better segregation of pseudohypoxic from non-pseudohypoxic tumors, confirming that pseudohypoxia and genuine hypoxia have differing gene expression signatures.

**Figure 2:**
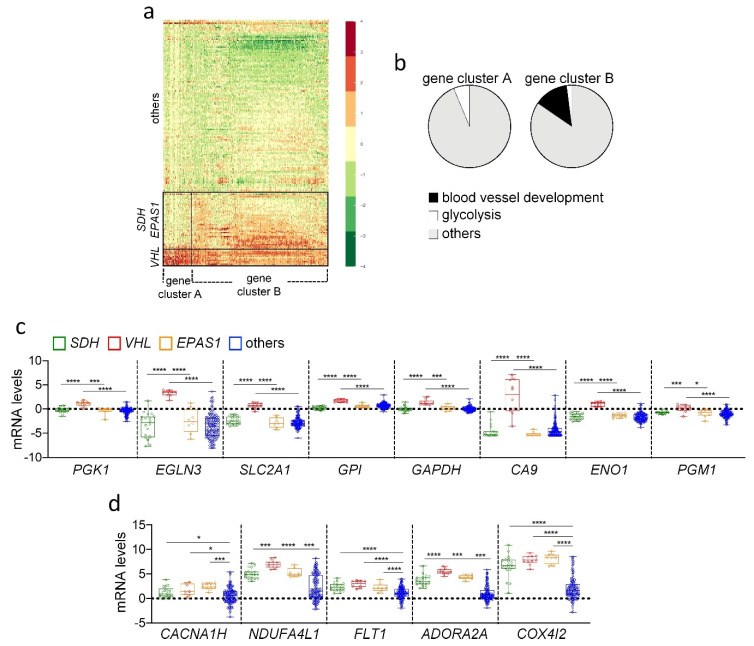
HIF2α- and HIF1α-target gene expression in PPGL. (**a**) Heatmap representation of the HIF-PPGL signature ranked by levels of gene expression. As shown, samples are clustered into three groups: *VHL*-PPGL; *SDH*/*EPAS1*-PPGL; others (PPGL lacking *VHL*, *SDH* or *EPAS1* mutations). All genes included in the HIF-PPGL signature can be clustered into two groups denoted as cluster A and B. (**b**) Graphic representation of the most enriched GO terms in the gene cluster A and B indicated in panel a. (**c**,**d**) The transcriptional levels of the indicated HIF1α (**c**) and HIF2α (**d**) target genes were analyzed in PPGL lacking (others) or carrying mutations in *SDH*, *VHL* or *EPAS1* genes using the TCGA. mRNA levels are expressed as Log-transformed mRNA z-scores compared to the expression distribution of all samples. Zero value is marked by a dashed line to better visualize differences among group of tumors. * *p* < 0.01, *** *p* < 0.001, **** *p* < 0.0001.

To get a better picture of the HIF-related transcriptome present in *SDH*/*EPAS1*- versus *VHL*-PPGL, a heatmap of the genes included in the HIF-PPGL signature was constructed (Figure 2A). This analysis confirmed the similarities among all pseudohypoxic PPGL and the differences between *VHL*-PPGL and *SDH*/*EPAS1*-PPGL. As shown, *VHL*-PPGL had a broader and stronger pseudohypoxic signature than *SDH*/*EPAS1*-PPGL, which included deregulation of glycolytic genes that were not overexpressed in *SDH*/*EPAS1*-PPGL (Figure 2A,B) (Supplementary Tables S5 and S6). Interestingly, targeted analysis of specific HIF1α- and HIF2α-responsive genes revealed that known HIF1α targets, such as *PGK1*, *EGLN3*, *SLC2A1*, *GPI*, *GAPDH*, *CA9*, *ENO1*, and *PGM1,* were strongly upregulated in *VHL*-PPGL in comparison with non-pseudohypoxic tumors, but were moderately upregulated or even unchanged in *SDH*- and *EPAS1*-PPGLs (Figure 2C). Conversely, HIF2α-related genes, such as *CACNA1H*, *NDUFA4L1*, *FLT1*, *ADORA2A* and *COX4I2,* were found upregulated in *VHL*-, *SDH*- and *EPAS1*-PPGLs (Figure 2D) as compared with non-pseudohypoxic tumors.

### 3.3. HIF2α Protein Expression in PPGL

The above data suggest that HIF2α rather than HIF1α is overexpressed in *SDH*-PPGL. At the protein level, there are few studies analyzing HIF2α in PPGL with differing results [40,41]. Moreover, the putative association of *SDH* mutations with HIFα protein accumulation has yielded contradictory results in cell lines [22,23,24]. Finally, besides the expected HIF2α localization at the cell nuclei, there are reports showing that this protein also accumulates in the cytoplasm [41,42] where it can regulate translation via non-transcriptional activity, as previously suggested [43,44,45]. Thus, we decided to perform immunohistochemical analysis of HIF2α in PPGL, analyze its relationship with the genotype of the tumors and pay attention to the subcellular distribution of the protein.

**Figure 3:**
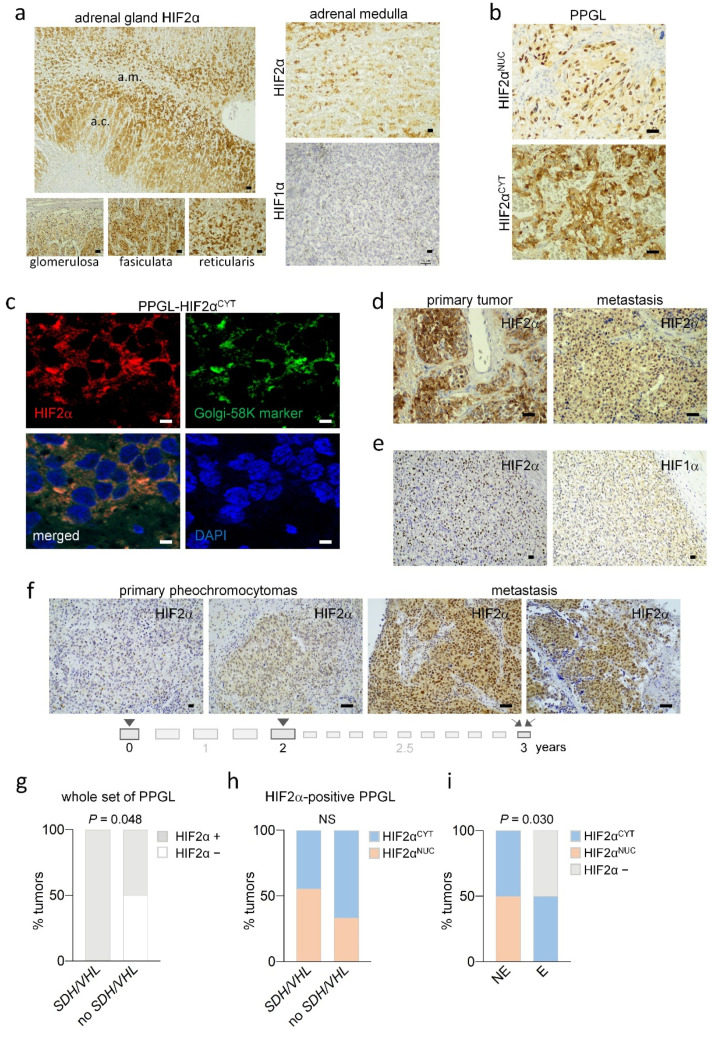
HIF2α protein expression in normal and tumoral PPGL. (**a**) Representative HIF2α and HIF1α immunohistochemical analysis in noncancerous AM. Images in the left panel correspond to the HIF2α immunostaining of the adrenal gland showing positive immunostaining at the adrenal cortex (a.c.) but not the medulla (a.m.). Detailed images of the zonas glomerulosa, fasciculata and reticularis of the adrenal cortex are also shown. Immunostaining of another noncancerous adrenal gland is shown in Supplementary Figure S2. (**b**) Representative images of HIF2α immunohistochemistry performed in PPGL showing nuclear (HIF2α^NUC^) or cytoplasmic (HIF2α^CYT^) immunostaining. (**c**) Representative images of double immunofluorescence with anti-HIF2α (red) and anti-Golgi-58K (green) in HIF2α^CYT^-PPGL. Cell nuclei were stained with DAPI (blue). (**d**) Representative images of HIF2α immunostaining performed in paired primary tumor and metastatic tissue. (**e**) Representative HIF2α and HIF1α immunostainings in a PPGL showing absence of HIF1α expression but intense HIF2α^NUC^ staining in the same tumor region. (**f**) Representative images of HIF2α expression in two PPGL primary tumors and their corresponding metastatic lesions. Time course of these events in years is shown below the pictures. (**g**) Percentage of tumors with HIF2α-positive (HIF2α+) or HIF2α-negative (HIF2α−) immunostaining classified according to the presence or absence of *SDH*/*VHL* mutations. (**h**) Percentage of HIF2α+ tumors with HIF2α^NUC^- or HIF2α^CYT^-positive immunostaining classified according to the presence or absence of *SDH*/*VHL* mutations. (**i**) Percentage of tumors with HIF2α-positive (HIF2α^NUC^ or HIF2α^CYT^) or HIF2α-negative (HIF2α−) immunostaining in norepinephrine (NE) or epinephrine (E) producing tumors. NS, not significant. Scale bars, 200 μm in upper picture in panel (**a**); 100 μm in the rest of panels.

To reliably assess expression in tumor tissues, we first asked whether this oxygen-sensitive protein accumulates in AM under physiological normoxic conditions. As shown in Figure 3A, HIF2α did not significantly accumulate in the nucleus of cells of the AM; only weak and diffuse staining was detected in the cytoplasm. We also checked HIF1α protein, which was not detected in this organ.

Immunohistochemical analysis of HIF2α was subsequently performed in 23 primary tumor samples obtained from 22 patients. HIF2α protein accumulation was detected in the tumor cell nuclei (HIF2α^NUC^, Figure 3B) in 39% of samples. Strong cytoplasmic (HIF2α^CYT^) immunostaining with granular appearance near the nuclei was found in 35% of tumors (Figure 3B). The remaining samples (26%) showed neither nuclear nor cytoplasmic immunostaining. Cytoplasmic HIF2α is likely located at the Golgi apparatus since double immunofluorescence of HIF2α and the Golgi-58K marker in HIF2α^CYT^-positively stained tumors indicated that both proteins co-localize (Figure 3C).

Regarding gene mutations, HIF2α-positive immunostaining was significantly more frequent in PPGL with *SDH* or *VHL* mutations than without (*p* = 0.048; Figure 3G). However, there were no associations with clinical or demographic features of patients (Supplementary Table S7). Both HIF2α^NUC^ and HIF2α^CYT^ immunostainings were detected in tumors with or without *VHL*/*SDH* mutations, although HIF2α^NUC^ immunostainings were slightly more frequent in *SDH*/*VHL*-PPGL than in tumors lacking these mutations (Figure 3H). We searched for somatic mutations in primary tumors with HIF2α^NUC^-positive immunostainings but lacking germline *SDH*/*VHL* mutations using our targeted gene-sequencing panel. However, we did not find any mutations or copy number variation (CNV) in the known PPGL-susceptibility genes.

Pseudohypoxic PPGL have been shown to favor over-production of norepinephrine rather than epinephrine, because of, among other factors, HIF2α-mediated repression of the *PNMT* gene involved in the conversion of norepinephrine to epinephrine. Comparison of HIF2α protein expression with the biochemical phenotype of tumors revealed that all norepinephrine-producing tumors had HIF2α-positive immunostaining, but it was not found in a preferential location of the staining at the nuclei or the cytoplasm of tumor cells (Figure 3I). Epinephrine-producing PPGL were either HIF2α negatives (50% of cases) or displayed HIF2α positivity located at the cytoplasm of the cells. Thus, HIF2α, and particularly nuclear HIF2α, is mostly, but not exclusively, accumulated in *SDH*/*VHL*-PPGL and norepinephrine secreting tumors.

### 3.4. HIF2α Protein Expression in Metastatic Tissues Derived from PPGL

HIF2α protein expression was also analyzed in seven metastatic lesions that had been surgically treated in five of the sixteen patients with “malignant” primary tumors carrying *SDHB* (*n* = 3) or lacking *SDH* or *VHL* (*n* = 2) mutations. In all cases, both, the “malignant” primary tumor and the matched metastasis had strong HIF2α immunostaining (Figure 3D).

Regarding HIF2α subcellular location and the presence or absence of *SDH*/*VHL* mutations, both the primary tumors and the metastases from patients with *SDHB* mutations were HIF2α^NUC^-positive, except for one case in which the metastasis derived from a primary PPGL with HIF2α^CYT^ immunostaining. In PPGL lacking *SDH*/*VHL* mutations, one metastasis had HIF2α^CYT^ immunostaining as its corresponding primary tumor. The other patient had developed two primary tumors that had been sequentially removed over a two-year period, and two metastatic lesions removed over one year after the last surgery. Intriguingly, HIF2α^NUC^ immunohistochemistry showed gradual increasing intensity of staining from the first to the second primary tumor and then to the two metastases (Figure 3F). The primary tumors of this patient did not have mutations in any of the PPGL-susceptibility genes. Thus, HIF2α^NUC^ positivity is frequent in metastatic tissues even when they arise from tumors lacking *SDH*/*VHL* mutations.

### 3.5. Comparison of HIF2α and HIF1α Protein Expression in PPGL

HIF2α-immunopositive cells, either HIF2α^NUC^ or HIF2α^CYT^, were homogeneously distributed throughout the whole tumor tissue, contrasting with the focal and patchy accumulation of HIF1α protein described in PPGL and most other types of cancers [21,46,47]. We thus evaluated HIF1α protein expression in 21 of the previously analyzed PPGL tumors, of which 76% were HIF2α-positive. We found that only 19% of those tumors had focally distributed HIF1α-positive cells in contrast to the homogeneous distribution of HIF2α. Figure 3E shows an example of a HIF2α^NUC^-positive/HIF1α-negative tumor. Thus, HIF1α and HIF2α expression was not correlated (*p* = 0.228, Supplementary Table S8), suggesting that different mechanisms operate upon HIF1α and HIF2α overexpression in PPGL.

### 3.6. EPAS1 mRNA Upregulation in Pseudohypoxic PPGL

**Figure 4:**
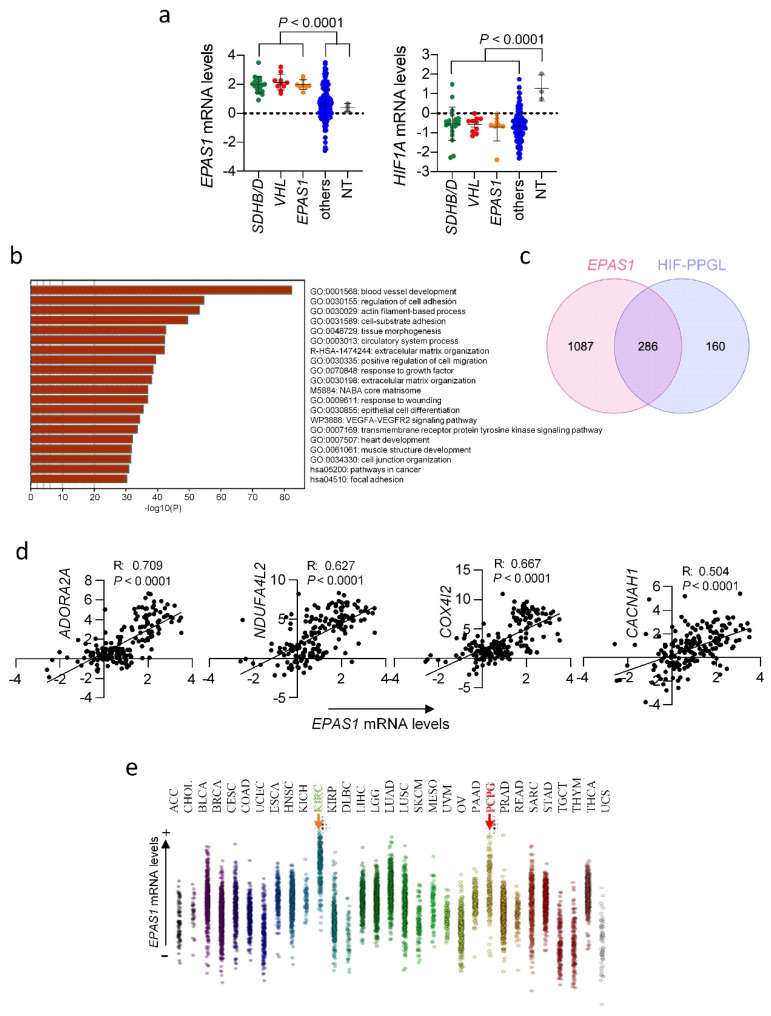
*EPAS1* and *HIF1A* mRNA expression in PPGL. (**a**) The transcriptional levels of *EPAS1* and *HIF1A* in PPGL lacking (others) or carrying mutations in *SDH*, *VHL* or *EPAS1* genes and in normal paraganglia tissues (NT) were analyzed based on the TCGA dataset. mRNA levels are expressed as Log-transformed mRNA z-scores compared to the expression distribution of all samples. Zero value is marked by a dashed line to better visualize differences among group of tumors. (**b**) Bar graph of top enriched terms across *EPAS1* co-overexpressed genes in PPGL, colored by *p*-values. (**c**) Venn diagram showing number of overlapping genes present in HIF-PPGL and *EPAS1*-associated signature. (**d**) Correlation of *EPAS1* mRNA levels with mRNA levels of *ADORA2A*, *NDUFA4L2*, *COX4I2* or *CACNAH1* using PPGL data included in TCGA project. (**e**) Comparative distribution of the *EPAS1* mRNA expression levels for several cancer types is represented with increasing expression levels from bottom to top in the *y*-axis (data obtained from the TCGA database). Tumors with the highest expression levels of *EPAS1* are indicated by colored arrows: KIRC (clear cell renal cell carcinoma) and PCPG (TCGA-code for PPGL). ACC, adrenocortical carcinoma; CHOL, cholangiocarcinoma; BLCA, bladder urothelial carcinoma; BRCA, breast invasive carcinoma; CESC, cervical squamous cell carcinoma and endocervical adenocarcinoma; COAD, colon adenocarcinoma; UCEC, uterine corpus endometrial carcinoma; ESCA, esophageal carcinoma; HNSC, head and neck squamous cell carcinoma; KICH, kidney chromophobe; KIRP, kidney renal papillary cell carcinoma; DLBC, lymphoid neoplasm diffuse large B-cell lymphoma; LIHC, liver hepatocellular carcinoma; LGG, brain lower grade glioma; LUAD, lung adenocarcinoma; LUSC, lung squamous cell carcinoma; SKCM, skin cutaneous melanoma; MESO, mesothelioma; UVM, uveal melanoma; OV, ovarian serous cystadenocarcinoma; PAAD, pancreatic adenocarcinoma; PRAD, prostate adenocarcinoma; READ, rectum adenocarcinoma; SARC, sarcoma; STAD, stomach adenocarcinoma; TGCT, testicular germ cell tumors; THYM, thymoma; THCA, thyroid carcinoma; UCS, uterine carcinosarcoma.

The analysis of the TCGA database revealed that all pseudohypoxic PPGL, including PPGL carrying *EPAS1* activating mutations, expressed higher levels of *EPAS1* mRNA than non-pseudohypoxic tumors or than normal sympathetic paraganglia. In contrast, *HIF1A* expression levels were lower in pseudohypoxic and non-pseudohypoxic PPGL than in normal tissues (Figure 4A). Correlation analysis of the *EPAS1* gene in PPGL revealed that tumors with high *EPAS1* expression levels had significant overexpression of genes involved in angiogenesis and blood vessel development, similarly to our findings in the HIF-PPGL profile (Figure 4B and Supplementary Table S9). In fact, 64% of the genes belonging to the HIF-PPGL signature (including *ADORA2A*, *NDUFA4L2*, *COX4I2* and *CACNA1H*) were significantly correlated with *EPAS1* expression levels (Figure 4C,D) suggesting that increased expression of *EPAS1* at the mRNA level may drive the pseudohypoxic signature of PPGL. Importantly, a pan-cancer analysis of *EPAS1* expression levels revealed that PPGL, together with ccRCC, are the human neoplasias with the highest mRNA levels of this gene (Figure 4E).

### 3.7. Absence of HIF2α Upregulation in HNPGL

**Figure 5:**
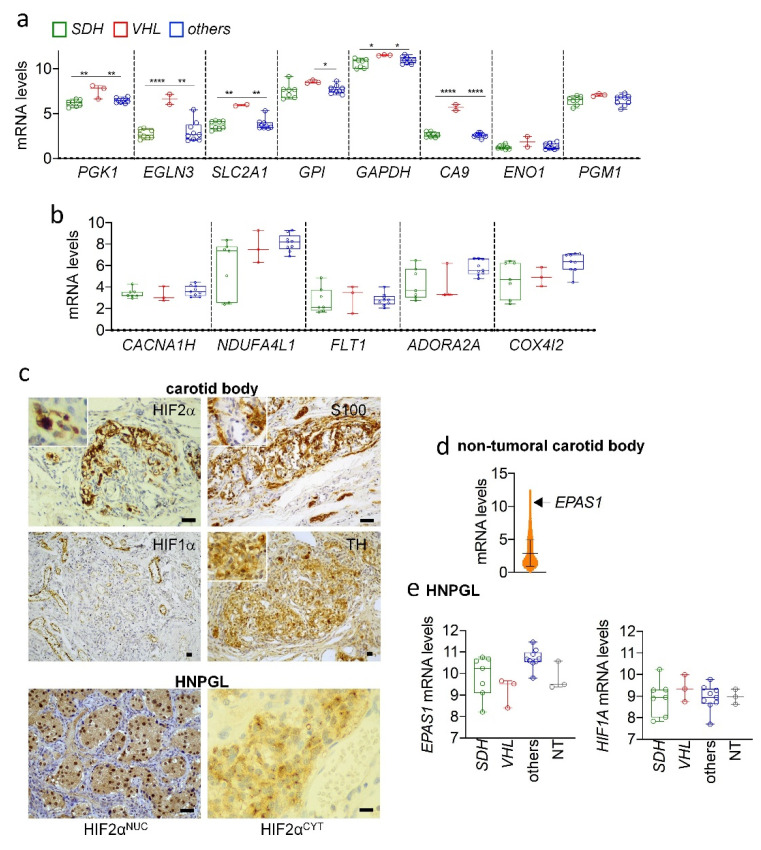
Non-tumoral and tumoral parasympathetic paraganglia express high levels of *EPAS1*/HIF2α. (**a**,**b**) The transcriptional levels of the indicated HIF1α (**a**) and HIF2α (**b**) target genes were analyzed in HNPGL lacking (others) or carrying mutations in *SDH* or *VHL* genes using the Affymetrix GeneChip Human Genome U133 Plus 2.0 Arrays [24]. (**c**) Representative images of HIF2α and HIF1α immunohistochemistry performed in non-tumoral CB and in HNPGL showing nuclear (HIF2α^NUC^) or cytoplasmic (HIF2α^CYT^) immunostaining. Immunostaining of S100, marker of sustentacular cells, and tyrosine hydroxylase (TH), marker of neuron-like glomus type I cells, are also shown. Insets show magnified images to better visualize glomus type I cells and sustentacular cells. Scale bars, 100 μm. (**d**) Distribution of the mRNA abundance of the whole genome gene expression data of non-tumoral CB obtained from the Affymetrix GeneChip Human Genome U133 Plus 2.0 Arrays [21]. The level of *EPAS1* mRNA is indicated by an arrow. (**e**) Transcriptional levels of *EPAS1* and *HIF1A* in HNPGL lacking (others) or carrying mutations in *SDH* or *VHL* and in normal CB (NT). * *p* < 0.01, ** *p* < 0.005, **** *p* < 0.0001.

To determine whether the HIF-related transcriptional pattern identified in PPGL was also present in HNPGL, we interrogated previously published whole-genome expression data in HNPGL [20]. This dataset has the advantage of including not only HNPGL with or without *SDH* mutations, but also HNPGL carrying somatic *VHL* mutations, which are highly infrequent in paraganglioma of parasympathetic linage. Similarly to our findings in PPGL, HIF1α-target genes such as *PGK1*, *EGLN3*, *SLC2A1* and *CA9* were more highly expressed in *VHL*-HNPGL than in *SDH*-HNPGL or HNPGL lacking *SDH* and *VHL* mutations (Figure 5A). Surprisingly, however, the mRNA levels of the HIF2α-related genes, *CACNA1H*, *NDUFA4L1*, *FLT1*, *ADORA2A* and *COX4I2*, were similar in all HNPGL, irrespective of whether they carried *SDH* or *VHL* mutations (Figure 5B).

HIF2α immunostaining was then performed in human non-tumoral CB and 32 HNPGL tumor tissues. In contrast to the non-tumoral AM, we found a strong HIF2α immunostaining at the chromaffin cells of the CB (Figure 5C, immunostainings of two independent sets of human carotid bodies are shown in Figure 5C and Figure S2). This agrees with our observation that *EPAS1* is among the most highly expressed genes in the CB (Figure 5D), likely explaining the high levels of HIF2α protein in this organ.

HIF2α immunohistochemical analysis of HNPGL revealed that 95% of tumors were positively stained, showing similar intensities as the normal CB. HIF2α^NUC^ immunostaining was present in 88% of cases; only 7% of samples displayed HIF2α^CYT^ staining located next to the nuclei (Figure 5C). The levels of HIF2α protein could not be quantified because protein extracts were not available for the tissue samples.

In contrast to PPGL, *EPAS1* mRNA was not found upregulated in pseudohypoxic HNPGL as compared with non-pseudohypoxic HNPGL or normal paraganglia (Figure 5E). There were no significant differences in HIF2α expression or in subcellular localization, irrespective of germline *SDH* mutations.

HIF1α immunohistochemistry performed in 30 of the abovementioned tissues revealed that 56% of samples were HIF2α- and HIF1α-positive and 36% were HIF2α-positive but HIF1α-negative. The remaining samples were both HIF2α- and HIF1α-negative. Thus, as in PPGL, HIF2α and HIF1α expression levels did not correlate in HNPGL (*p* = 0.179, Supplementary Table S8).

### 3.8. Succinate Induces Overexpression of COX4I2, NDUFA4L2 and ADORA2A in a HIF2α-Dependent Manner

**Figure 6:**
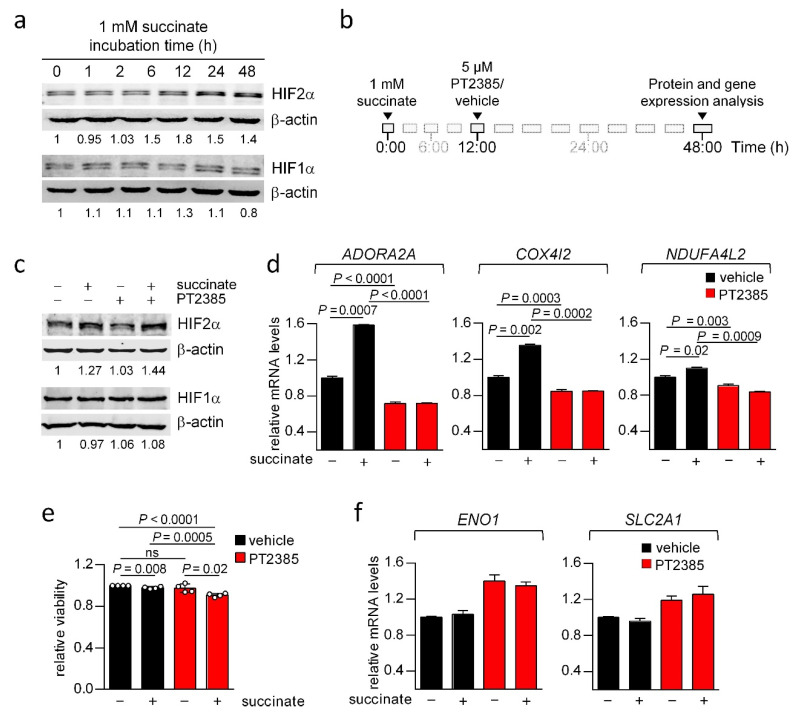
Induction of *ADORA2A*, *COX4I2* and *NDUFA4L2* expression by succinate is HIF2α-dependent. (**a**) Representative immunoblots of HIF2α and HIF1α in PC12 cells treated with 1 mM succinate for the indicated time. β-actin was used to assess equal loading. Numbers represent the relative expression of HIF2α and HIF1α (**b**,**c**). PC12 cells were preincubated with 1 mM succinate for 12 h before the addition of 5 μM PT2385 (or vehicle) and subsequent incubation for 36 h, as outlined in (**b**). A representative immunoblot showing HIF2α and HIF1α protein levels in the different conditions is shown in panel (**c**). β-actin was used to assess equal loading. Numbers represent the relative expression of HIF2α and HIF1α. (**d**) Relative mRNA levels of *ADORA2A*, *COX4I2* and *NDUFA4L2* in PC12 cells treated or untreated with succinate in the presence or absence of 5 μM PT2385. (**e**) Cell viability was determined by CellTiter 96^®^ AQueous One Solution Cell Proliferation Assay in PC12 cells treated or untreated with succinate in the presence or absence of 5 μM PT2385. (**f**) *ENO1* and *SLC2A1* mRNA levels in PC12 cells treated or untreated with succinate in the presence or absence of 5 μM PT2385. PT2385 vehicle: 0.05% DMSO. Uncropped Western Blots can be found at supplementary original images.

The above data suggest that high succinate levels induce expression of HIF2α- rather than HIF1α-target genes. A previous report showed that *sdhb*−/− immortalized mouse chromaffin cells express high levels of HIF2α protein [48]. In addition, expression of *COX4I2*, *NDUFA4L2* and *ADORA2A* have been shown to be HIF2α-dependent in mouse CB and rat sympathoadrenal cells [8,10]. Here, we sought to determine whether an increase in extracellular succinate levels is able to induce HIF2α protein accumulation in physiological oxygen tensions and increase expression of *ADORA2A*, *COX4I2* and *NDUFA4L2* in the context of a tumor background. To this end, we used PC12 cells derived from a rat pheochromocytoma lacking *SDH* mutations and incubated them in the presence (or absence, as a control) of 1 mM succinate. As shown in Figure 6A, succinate increased HIF2α levels over time (1.8-fold increase at 12 h of exposure), whereas it had a marginal and transient effect on HIF1α protein (Figure 6A, 1.3-fold increase at 12 h of exposure). Induction of HIF2α but not that of HIF1α has been also demonstrated in neuroblastoma cells [48]. Succinate also induced expression of *ADORA2A*, *COX4I2*, and *NDUFA4L2* and (1.35-, 1.1- and 1.6-fold change, respectively) (Figure 6D). These effects were blunted by PT2385 (5 μM), a specific inhibitor of HIF2α transcriptional activity (Figure 6B,D). PC12 cells accumulate low amount of HIF2α under normoxic conditions. In line with this, PT2385 also repressed *ADORA2A*, *COX4I2* and *NDUFA4L2* expression in cells that had not been exposed to succinate. PT2385 interferes with the assembly of HIF2α/HIF1β heterodimers acting as an inhibitor of the transcriptional activity of HIF2α that may or not affect its protein levels [49]. Treatment of PC12 cells with PT2385 in the presence or absence of succinate did not modify HIF2α or HIF1α protein levels (Figure 6C). In addition, PT2385 treatment (36 h exposure to 5 μM PT2385) had a very mild effect on cell viability (Figure 6E), thus ruling out a major toxic effect under our experimental conditions.

To rule out the possibility that the effects of succinate and PT2385 were dependent on HIF1α, two HIF1α-target genes (*ENO1* and *SLC2A1*) were analyzed in PC12 cells exposed to succinate in the presence or absence of the HIF2α inhibitor (Figure 6F). As shown, succinate did not induce enhancement of *ENO1* or *SLC2A1* mRNA levels. In addition, PT2385 did not reduce *ENO1* or *SLC2A1* mRNA levels in cells, irrespective of their exposure to succinate.

We also verified that induction of *ADORA2A*, *COX4I2* and *NDUFA4L2* by hypoxia or overexpression of the normoxically stable mutated (P405A/P531A) HIF2α protein was dependent on HIF2α activity (Supplementary Figure S3). By contrast, expression of *ENO1* and *SLC2A1* increased in PC12 cells exposed to hypoxia, but this was not prevented by PT23815. Similar results were obtained by overexpression of the P405A/P531A mutant HIF2α. Exposure of PC12 cells to PT2385 reduced cell viability of hypoxic PC12 cells, but this effect was negligible (Supplementary Figure S3). The succinate and hypoxia effects on HIF2α activity were replicated in a different cell line derived from squamous cell carcinoma (SCC38 cells) (Supplementary Figure S4).

Collectively, these data show that succinate-induced *ADORA2A*, *COX4I2* and *NDUFA4L2* expression is dependent on the HIF2α-transcriptional activity, but not on HIF1α-transcriptional activity.

## 4. Discussion

We report here a detailed comparative analysis of HIF2α protein expression and HIF-related transcriptome in HNPGL and PPGL, which provides some clues on the role of this transcription factor in the development of these tumors.

For over 10 years, a fundamental question regarding the association of *SDH* mutations and HIF1α or HIF2α activation has been a subject of debate, and through a variety of research methods, conflicting evidence has accumulated [22,23,24]. In the case of PPGL, there are studies that provide some support for the theory on the impact of global HIF (HIF1α and HIF2α) regulation in *SDH-* and *VHL*-related tumors [50,51], while others advocate for a role of only one of the HIFα subunits [48]. There are also reports that either do not replicate previously reported data or contradict them [20,21]. Most previous reports have performed gene expression studies not accompanied by protein expression data, did not analyze normal tissues, or did not make comparisons between sympathetic and parasympathetic paragangliomas. Herein, we aimed at covering all these understudied subjects and providing protein expression data, which could have relevant implications not only in the field of tumor biology, but also in the field of hypoxia-sensing in humans.

First, we provided novel data on HIF2α protein expression in noncancerous human CB and AM, relevant for the understanding of the role of this protein in tumorigenesis. Our data show that HIF2α accumulates at high levels in the chromaffin cells of human CB under physiological conditions, but HIF1α does not. Moreover, *EPAS1* was found to be one of the most highly expressed genes in this organ, thus suggesting that high protein synthesis may saturate the capacity of the cells to degrade HIF2α at physiological oxygen levels. HIF2α is known to be stabilized in endothelial cells and macrophages and highly expressed in the lung, heart, placenta and kidney [52], but until now, its expression in human paraganglionic cells was unknown. Our data are partially consistent with previously reported findings in mice [6,8], although those studies did not demonstrate that high levels of *EPAS1* mRNA were translated into high protein levels, which could have been considered doubtful because of the oxygen-sensitive nature of the protein. In contrast to the CB, chromaffin cells of the human noncancerous AM did not significantly accumulate HIF2α, thus implying that HIF2α has a differential role in ganglia of sympathetic versus parasympathetic origins.

We also showed that HIF2α protein and target genes are not overexpressed in HNPGL in comparison with CB, thus casting doubts as to whether this transcription factor confers tumorigenic advantage to this system. Previous studies in mouse models revealed that *EPAS1* over-activation may confer survival advantage to CB chromaffin cells and induce hypertrophy of this paraganglia [6,7]. However, this was proven to be insufficient to induce tumor development. Moreover, *EPAS1* gain-of-function mutations have been found in PPGLs [16,51], but to the best of our knowledge, they have not been identified in HNPGL, thus illustrating again the putative differential role of HIF2α in the sympathetic and parasympathetic system.

PPGL have been more extensively studied in the past than HNPGL. However, there has been no clear evidence of the role of HIF1α and/or HIF2α in *SDH*-mutated tumors. We show here that HIF2α protein is preferentially accumulated in tumor cells of *SDH*-mutated PPGL, but HIF1α is not. Importantly, we also showed that the HIF2α-related transcriptome prevails over that of HIF1α in *SDH*-mutated PPGL in comparison with PPGL lacking those mutations, or PPGL carrying mutations in other genes involved in PPGL pathogenesis. Given that HIF2α target genes are tissue-type-specific, we highlighted that the HIF2α gene expression module identified here contains those genes known to be regulated by HIF2α in neural-crest derived chromaffin cells. The HIF2α-related transcriptional module was also identified in *VHL*-mutated PPGL, although these types of tumors, in contrast to *SDH*-mutated PPGL, also overexpress HIF1α-target genes.

These data could have important clinical implications. Pharmacological strategies targeting HIF2α- or HIF2α-signaling pathways would be beneficial for all patients with pseudohypoxic PPGL, whereas drugs targeting HIF1α- or HIF1α-mediated metabolic switches would more efficiently benefit patients with *VHL*-mutated PPGL. Importantly, we found that metastatic tissues express high levels of nuclear HIF2α, even when the tumor arises from a PPGL lacking mutations in any of the oxygen-sensing related genes associated with PPGL tumorigenesis, thus suggesting that metastatic PPGL could be benefited by therapies targeting HIF2α. Belzutifan, a potent and selective small-molecule inhibitor of HIF2α analog of PT2385, has proven to be of remarkable therapeutic benefit for patients with ccRCC and the Pacak-Zhuang syndrome caused by loss-of-function mutations in *VHL* or gain-of-function mutations in *EPAS1*, respectively [25,26]. Based on our data, we suggest that the analysis of HIF2α protein expression would be relevant to selecting patients susceptible to treatment with this drug.

We also provide here a detailed map of the in vivo genetically determined pseudohypoxic signatures, which differ from the hypoxia-induced transcriptome in cancer. Genetically induced pseudohypoxia elicits an angiogenic phenotype, whereas genuine hypoxia mainly induces metabolic reprograming. Several HIF2α-related genes identified in our analysis could have important implications in the pathophysiology of PPGL. On one hand, *COX4I2* encodes a tissue-specific mitochondrial complex IV subunit, and it is expressed in a HIF2α- and succinate-dependent manner in rat chromaffin cancer cells and in PPGL. Recently, *COX4I2* has been reported to be essential for oxygen-sensing functions of CB and lung, and for inducing aberrant reactive oxygen species (ROS) production [8,53]. Redox imbalance has been implicated in multiple aspects of cell physiology and cancer pathology, such as phenotypic plasticity and disrupted differentiation. Therefore, *COX4I2* could be a relevant, not previously recognized, mediator of HIF2α signaling in PPGL. Another gene identified here, in association with succinate and HIF2α activity, is *ADORA2A* encoding the A2a receptor of adenosine. Brown et al. showed that *ADORA2A* is overexpressed in response to hypoxia via HIF2α, and contributes to catecholamine secretion in developing chromaffin cells [54]. On another hand, overexpression of this gene has been associated with protection against immune attack in cancer, and as a consequence, compounds inhibiting A2a receptors are being tested for cancer immunotherapy [55]. Those inhibitors could be beneficial for the treatment of patients with pseudohypoxic PPGL. Overall, these findings should be considered when choosing therapeutic strategies targeting HIFα subunits or HIF-signaling pathways in PPGL.

Paraganglioma with high levels of HIF2α do not accumulate HIF1α, and vice versa, suggesting that the two subunits undergo distinct deregulatory pathways. Accumulation of HIF2α in pseudohypoxic-PPGL and in all HNPGL is paralleled by high levels of *EPAS1* mRNA. In fact, PPGL, together with ccRCC, are the tumor types with the highest levels of *EPAS1* mRNA. In addition, PPGL with high expression levels of *EPAS1* mRNA also overexpress genes involved in angiogenesis, many of which are also present in the HIF-PPGL signature. Collectively, these data suggest that, in contrast to HIF1α, HIF2α may be regulated not only post-translationally in PPGL, but also at the mRNA level. Nevertheless, HIF2α is known to be expressed in migrating neural-crest-derived cells in human embryos, where it is regulated by both oxygen-dependent and oxygen-independent mechanisms [56,57]. Thus, another possibility is that, as previously suggested [58], increased expression of HIF2α in PPGL is not the result of deregulation of *EPAS1* gene expression but rather the consequence of a selective increased growth or survival of immature HIF2α-expressing neural crest cells that remain in the adult paraganglia.

We also showed that HIF2α has a dual nuclear or cytoplasmic localization that is mutually exclusive, similar to what has been observed in neuroblastoma and glioblastoma cells or renal epithelial cells [44,59]. However, we did not observe preferential nuclear or cytoplasmic location in relationship with gene mutation or biochemical phenotype of tumors. Although the functional significance of the distinct subcellular localization of HIF2α remains unknown, our data indicate that it is plausible that HIF2α has noncanonical functions in PPGL. Uniacke et al. reported that HIF2α complexes with RBM4 and eIF4E2 in the cytoplasm, mediating selective cap-dependent translation [60]. We showed that HIF2α^CYT^ was near the nuclei of the tumor cells and co-localized with the Golgi-58K marker. Thus, further investigations should analyze canonical and noncanonical HIF2α activities, since the HIF2α function in PPGL may be more complex than expected.

## 5. Conclusions

In summary, our observations showed for the first time that HIF2α seems to have a differential role in paraganglia of sympathetic versus parasympathetic origins under physiological and tumoral conditions. HIF2α is highly expressed in CB, and is not deregulated in HNPGL. By contrast, HIF2α is the dominant HIFα factor influencing the pseudohypoxic transcriptome in *SDH*-mutated PPGL. We also showed that the activity of both HIF1α and HIF2α contributes to the pseudohypoxic transcriptome of *VHL*-PPGL. These observations have far-reaching implications, given that they support the hypothesis that the therapeutic strategies targeting HIF2α and/or HIF2α-responsive genes may be relevant for the successful treatment of patients with pseudohypoxic PPGL.

## Data Availability

The data presented in this study are available in this article and the Supplementary Materials.

## Supplementary Materials

The following supporting information can be downloaded at: https://www.mdpi.com/article/10.3390/cancers14122986/s1, Table S1: Clinical data of patients; Table S2: Correlation matrix for 10 HIF-related genes in PPGL; Table S3: List of genes included in the HIF-PPGL signature and GO/pathway enrichment; Table S4: Genes overlapping in HIF-PPGL and other hypoxia-related signatures; Table S5: List of genes contained in the HIF-PPGL signature that are commonly upregulated in *VHL* and *SDH*/*EPAS1*-PPGL, GO/pathway enrichment; Table S6: List of genes contained in the HIF-PPGL signature that are upregulated in *VHL*-PPGL, GO/pathway enrichment; Table S7: Association of HIF2α immunostaining and genetic and clinical data; Table S8: Association of HIF1α and HIF2α expression; Table S9: List of genes positively correlated with *EPAS1* in PPGL and GO/pathway enrichment; Figure S1: Analysis of the specificity of HIF2α antibody; Figure S2: HIF2α immunostaining of human adrenal gland and carotid body; Figure S3: Induction of *ADORA2A*, *COX4I2* and *NDUFA4L2* expression by hypoxia is HIF2α-dependent in PC12 cells. Figure S4: Induction of HIF2α expression by succinate in cells derived from squamous cell carcinoma. Figure S5: Matrix of all pairwise intersections.

## Appendix A

### Appendix A.1. Selection of 10 HIF-Target Genes for Identification of HIF-PPGL Signature

To define the HIF-PPGL signature, we first sought to identify the set of genes that best define the canonical hypoxic and HIF associated genetic profile in cancer. miR-210, one of the most reliable biomarkers of hypoxia and pseudohypoxia, was selected to identify genes that were jointly overexpressed with the microRNA in each of the 22 cancer types included in the TCGA datasets. Positively correlated genes (*R* > 0.4, adjusted *p*-value < 0.01; Pearson’s correlation) were selected and then intersected to obtain a set of overlapping genes in cancer. A total of 138 genes were commonly co-expressed with miR-210 in ≥10 types of cancers. This included *P4HA1*, *LDHA*, *BNIP3*, *CA9*, *PGK1*, *SLC2A1*, *ENO1*, *ALDOA*, *TPI1* and *GAPDH*, which were used as baits to identify the HIF-PPGL signature.

### Appendix A.2. In Silico Gene Expression Analysis

The TCGA database of paragangliomas (184 primary tumors) included 33 pseudohypoxic PPGL, defined here as those carrying mutations in *VHL* (seven PPGL with germline mutations and three PPGL with somatic mutations), *SDH* (twelve PPGL with germline *SDHB* mutations and three PPGL with *SDHD* mutations) or *EPAS1* (eight PPGL with somatic mutations) genes. The remaining tumors did not carry *VHL*/*SDH*/*EPAS1* mutations, and were thus considered as non-pseudohypoxic. mRNA expression levels of HIF-related genes are presented as log2 (x + 1)-transformed RNA-Seq by Expectation-Maximization (RSEM) normalized count.

Expression of HIF-related genes (*P4HA1*, *LDHA*, *BNIP3*, *CA9*, *PGK1*, *SLC2A1*, *ENO1*, *ALDOA*, *TPI1* and *GAPDH*) was correlated with the whole RNAseq data. Positively correlated genes in each of the 10 gene sets (*R* > 0.4, adjusted *p*-value < 0.01; Pearson’s correlation) were selected (the full correlation matrix is provided in Supplementary Table S2) and then intersected to obtain a set of overlapping genes (see pairwise intersections in Supplementary Figure S5).

Rooted in Weber’s law, aligned spatial position has been ranked as one of the most effective visual channels to express ordered magnitudes [28] and the one that allows for the most accurate perception of relative positions [29], and thus is also effective for comparing distributions. We embraced this design principle in Figure 4E, where *EPAS1* mRNA expression levels for several cancer types are represented sharing the same scale in the *y*-axis, with increasing expression levels from bottom to top.

Additionally, t-SNE [30] was used for visualization of the local structure of the gene expression levels of the high-dimensional data. The t-SNE dimensionality reduction algorithm maps a set of high-dimensional samples $x_{i}\in {\mathbb{R}}^{D}$ to a set of low-dimensional points $y_{i}\in {\mathbb{R}}^{d}$, maximizing the chance that two neighbor samples $x_{i},x_{j}$ in the input space are mapped to points $y_{i},y_{j}$, which are also neighbors in the low-dimensional space. This is achieved by minimizing the Kullback–Leibler distance KL(P∥Q) between the joint probability distributions P,Q based on the conditional probabilities of $x_{i}$ being the neighbor of $x_{j}$ and $y_{i}$ being the neighbor of $y_{j}$. In the setup for gene expression analysis, d is set to 2, resulting in a 2D scatter plot where each point represents a biological sample defined by a large number D of gene expression attributes, and two close points in the visual map correspond to biological samples with similar gene expression patterns.

Part of the data analysis pipeline was carried out using a visual analytics tool featuring the Morphing Projections (MP) technique [31]. MP allowed smooth switching between different 2D scatterplots of the samples, with spatial arrangements based on different criteria. In our study, this included the t-SNE projections of gene expression patterns (Figure 1E), which could be combined with specific layouts revealing clinical information, such as vertical arrangements by cancer types and horizontal arrangements of expressions of a particular gene; for instance, *EPAS1* (see Figure 4E).

Previously reported whole genome relative gene expression data of HNPGL obtained with the Affymetrix GeneChip Human Genome U133 Plus 2.0 Arrays was used to compare the expression of HIF-related genes [21]. This set of samples included three non-tumoral CB, three tumors carrying somatic *VHL* mutations, six tumors from patients with *SDHB* or *SDHD* germline mutations and nine tumors lacking mutations germline or somatic mutations in the *SDH* or *VHL* genes.

GO and pathway enrichment analyses were performed with Metascape [61].

### Appendix A.3. Cell Treatments

The established human squamous cell carcinoma (SCC)-derived cell line SCC38 was kindly provided by Dr. Reidar Grenman (University Central Hospital, Turku, Finland). The cells were grown in Dulbecco’s modified Eagle’s medium supplemented with 10% fetal bovine serum, 100 U/mL penicillin and 100 μg/mL streptomycin. Succinate and PT2385 treatments were performed as described for PC12 cells. For hypoxic treatments, cells at 70–80% confluence were either exposed to continued normoxia or placed in a hypoxic incubator (HeraCell150) that maintained a constant environment (37 °C, 5% CO_2_ and 1% O_2_ balanced with N_2_). Transient transfections with pBabe-puro as control or HA-HIF2alpha-P405A/P531A-pBabe-puro, a gift from William Kaelin [62] (Harvard Medical School, Boston, MA, USA), were performed with Lipofectamine LTX (Thermo Fisher Scientific, Waltham, MA, USA) according to the manufacturer’s protocol, at a final concentration of 2.5 µg of plasmid DNA.
