# Supplementary material for: Differential HIF2α Protein Expression in Human Carotid Body and Adrenal Medulla under Physiologic and Tumorigenic Conditions
Source: Cancers (Basel). 2022 Jun 17;14(12):2986. doi: 10.3390/cancers14122986 (PMC9221385; doi:10.3390/cancers14122986)
Supplement: Supplementary file 1 [file cancers-14-02986-s001.zip › cancers-1766034-supplementary.pdf]

*Supplementary Information*

# Differential HIF2α protein expression in human carotid body and adrenal medulla under physiologic and tumorigenic conditions

Lucía Celada, Tamara Cubiella, Jaime San-Juan-Guardado, Andrés San José Martínez, Nuria Valdés, Paula Jiménez-Fonseca, Ignacio Díaz, Jose María Enguita, Aurora Astudillo, Enol Álvarez-González, Luisa María Sierra and María-Dolores Chiara

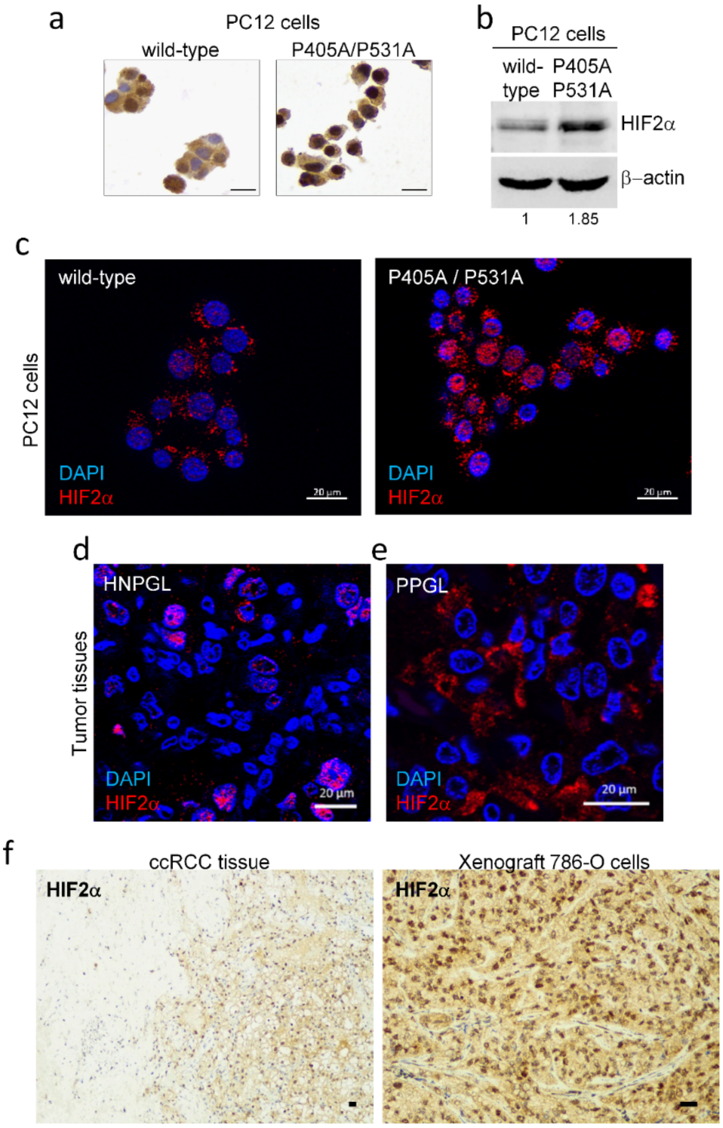


**Figure S1.** Analysis of the specificity of HIF2α antibody. (**a**—**c**) PC12 cells were transiently transfected with plasmids expressing wild-type HIF2α or normoxically stable P405A/P531A mutant HIF2α. Expression of HIF2α was then analyzed by immunocytochemistry (counterstained with hematoxylin) (**a**), western blot (**b**) or immunofluorescence (**c**). Numbers below western blot represent the relative expression of HIF2α As shown in panels **a** and **c**, higher expression levels of nuclear HIF2α was detected in cells transfected with normoxically stable P405A/P531A mutant HIF2α than with wild-type HIF2α. (**d**–**e**). Representative images of HIF2α immunofluorescence in HNPGL (**d**) and PPGL tumor tissues (**e**). (**f**) Representative images of HIF2α immunohistochemistry in a clear cell renal cell carcinoma (ccRCC) tissue or a mouse xenograft produced by inoculation of the *VHL*-mutant ccRCC-derived 786-O cells. Note that HIF2α is detected at the cell nuclei of HNPGL and ccRCC samples but not in PPGL which showed cytoplasmic perinuclear staining. Scale bars, 20 µm (**c**, **d**, **e**), 50 µm (**a**), 100 µm (**f**).

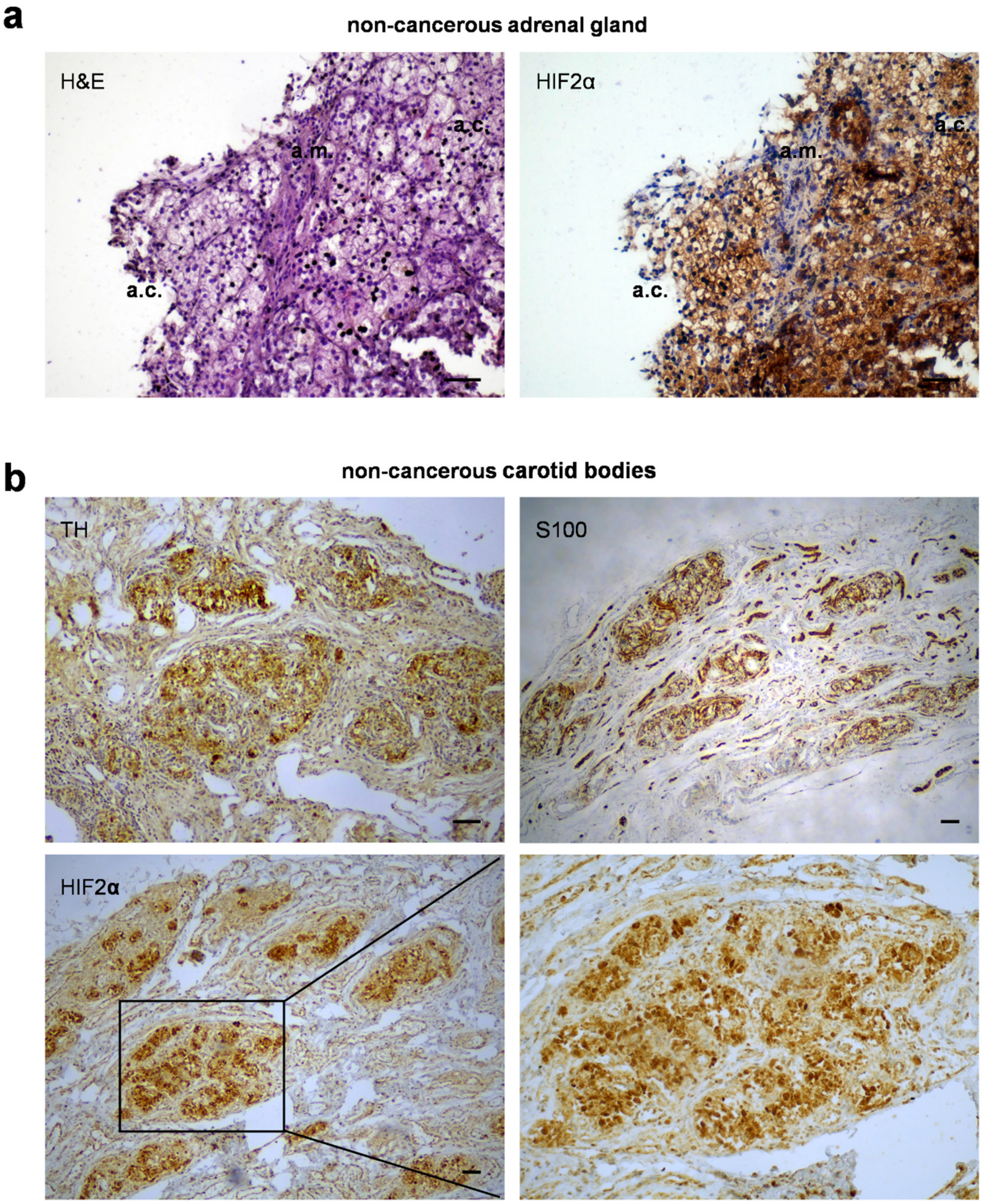


**Figure S2. HIF2α immunostaining of human adrenal gland and carotid body.** (**a**) Representative images of hematoxylin-eosin (H&E) staining and HIF2α immunohistochemistry performed in non-cancerous human adrenal medulla. a.c., adrenal cortex; a.m., adrenal medulla. (**b**) Representative TH, S100 and HIF2α immunostainings in a non-tumoral human carotid body. Scale bars, 50 μm.

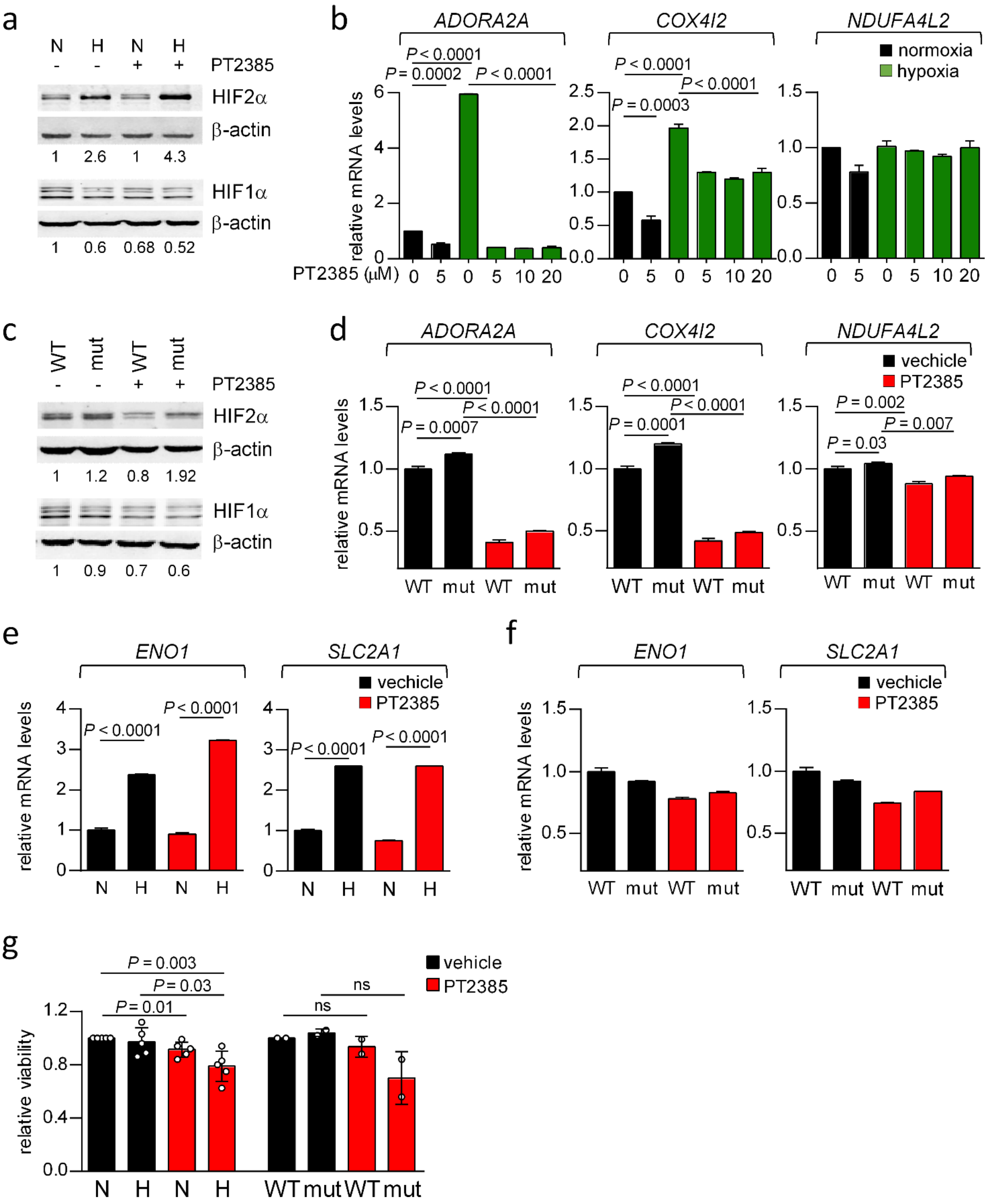


**Figure S3. Induction of *ADORA2A*, *COX4I2*, and *NDUFA4L2* expression by hypoxia is HIF2α-dependent in PC12 cells. (a–f).** Representative HIF2α and HIF1α immunoblots (a, c) and mRNA levels of the indicated genes (**d**, **e**, **f**) in PC12 cells exposed (H) or not (N) to hypoxia (1% $O_2$) for 36 h, or transiently transfected with plasmids encoding wild–type HIF2α (WT) or P405A/P531A mutant HIF2α (mut). PT2385 (or vehicle: 0.05% DMSO) was added to the cell culture at 5 μM final concentration . Time of exposure to PT2385 was 30 h. β-actin was used to assess equal loading. Numbers below immunoblots represent the relative expression levels of each protein. (**b**). Relative mRNA levels of the indicated genes in PC12 cells exposed or not to hypoxia (1% oxygen) for 36 h in the absence or presence of the indicated concentrations of PT2385. (**g**). Cell viability was determined by CellTiter 96® AQueous One Solution Cell Proliferation Assay in PC12 cells treated or not with 5 μM PT2385 for 30h. PT-2385 treatment was performed under normoxic (N) or hypoxic (H, 1% oxygen) conditions or in cells transfected with plasmids encoding wild–type (WT) HIF2α or P405A/P531A mutant (mut) HIF2α ns: not significant. Uncropped Western Blots can be found at supplementary original images.

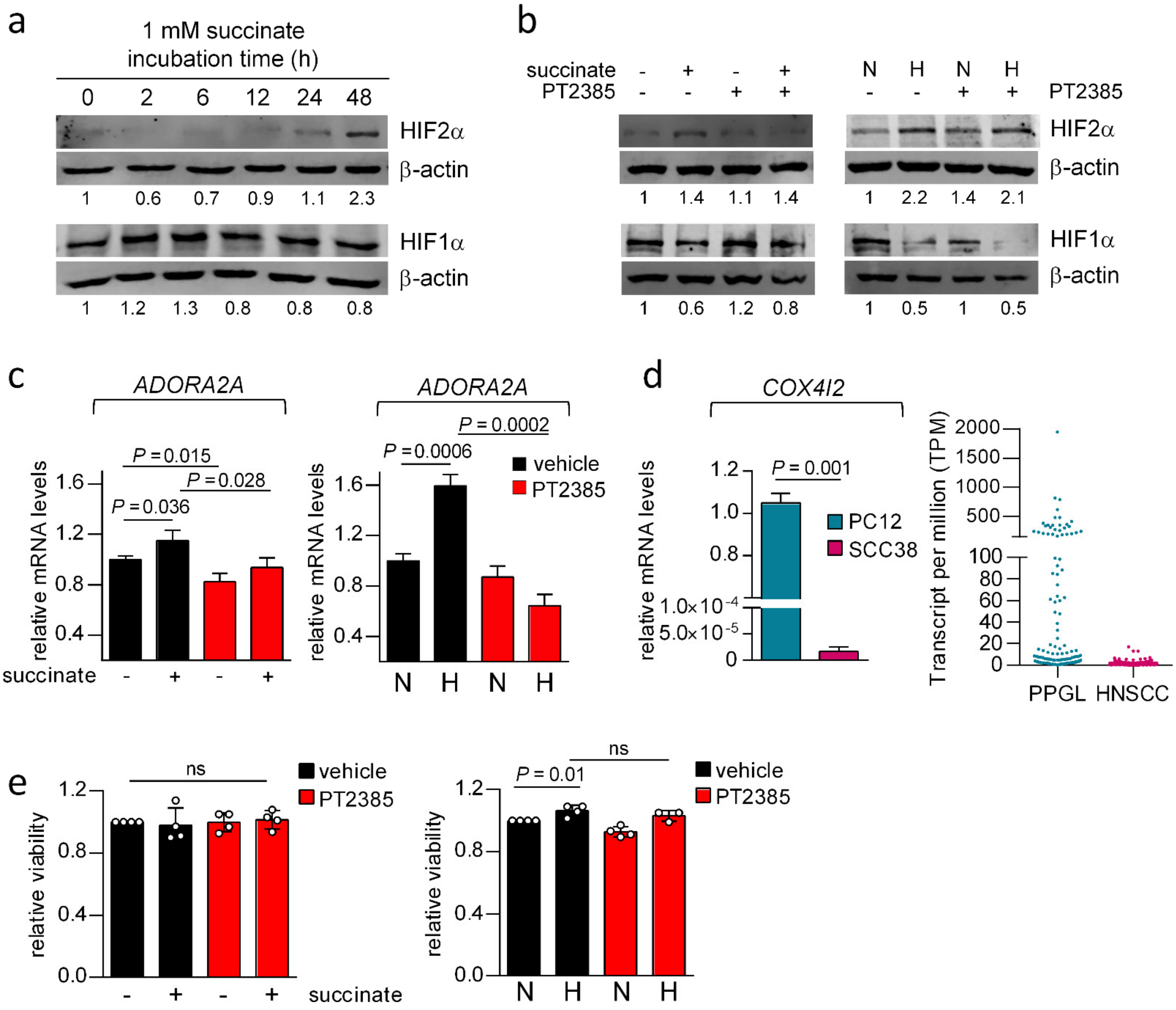


**Figure S4. Induction of HIF2α expression by succinate in cells derived from squamous cell carcinoma.** (**a**) Representative immunoblots of HIF2α and HIF1α in SCC38 cells treated with 1 mM succinate for the indicated time (h). (**b, c, e**) SCC38 cells were preincubated with 1 mM succinate (or vehicle) for 12 h or exposed or not (N) to 1% $O_2$ (H) for 6 h before the addition of 5 µM PT2385 (or vehicle: 0.05% DMSO) and subsequent incubation for 36 h. A representative immunoblot showing HIF2α and HIF1α protein levels in the different conditions is shown in panel **b**. β–actin was used to assess equal loading. Numbers represents the relative expression of HIF2α and HIF1α. (**c**) Relative mRNA levels of *ADORA2A* in SCC38 cells. (**d**) Relative mRNA levels of *COX4I2* in PC12 and SCC38 cells showing the almost negligible levels of this gene in SCC38 cells as compared with PC12 cells (left panel). *COX4I2* expression levels were also evaluated in tumors using the TCGA database of head and neck squamous cell carcinoma (HNSCC) and PPGL (right panel). As shown, *COX4I2* gene is highly expressed in PPGL but not detected in most HNSCC. Therefore, *COX4I2* gene expression could not be evaluated in SCC38 cells. (**e**). Cell viability was determined by MTS assay in SCC38 cells treated as indicated. **.** Uncropped Western Blots can be found at supplementary original images.

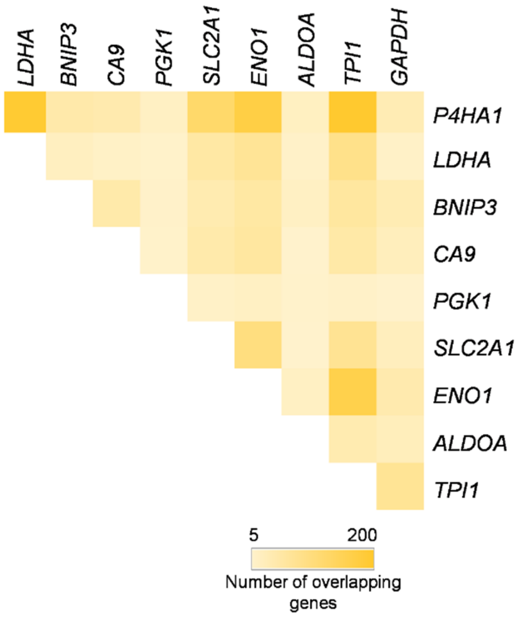


**Figure S5. Matrix of all pairwise intersections.** Minimal triangular matrix showing pairwise intersections of the 10 gene sets obtained through the analysis of positive correlations with *LDHA, BNIP3, CA9, PGK1, SLC2A1, ENO1, ALDOA, TPI1* and *GAPDH*.

**Table S1.** Clinical data of patients.

| | | Primary tumor type | |
|---|---|---|---|
| | | PPGL | HNPGL |
| Tumor location | | | |
| | extradrenal | 9 | |
| | adrenal | 14 | |
| | carotid body | | 15 |
| | vagal PGL* | | 3 |
| | jugulo-tympanic PGL* | | 13 |
| | parapharynx | | 1 |
| | metastasis | 6 | 1 |
| Gender | | | |
| | female | 9 | 24 |
| | male | 14 | 8 |
| Age at diagnosis (years): mean (range) | | 41.7 (14-83) | 46.7 (25-73) |
| Metastatic disease | | | |
| | no | 9 | 31 |
| | yes | 14 | 1 |
| Genotype | | | |
| | *SDHB* | 8 | 4 |
| | *SDHD* | 0 | 11 |
| | *SDHC* (epimutation) | 0 | 1 |
| | *VHL* | 1 | 1 |
| | *RET* | 1 | 0 |
| | *NF1* | 1 | 0 |
| | *SDH/VHL/EPAS1* WT | 12 | 15 |

*paraganglia

**Table S2.** Correlation matrix for 10 HIF-related genes in PPGL.

Supp_Table S2.xlsx

**Table S3.** List of genes included in the HIF-PPGL signature and GO/pathway enrichment.

Supp_Table S3.xlsx

**Table S4.** Genes overlapping in HIF-PPGL and other hypoxia-related signatures.

Supp_Table S4.xlsx

**Table S5.** List of genes, contained in the HIF-PPGL signature, that are commonly upregulated in *VHL* and *SDH*/*EPAS1*-PPGL. GO/pathway enrichment.

Suppl Table S5.xlsx

**Table S6.** List of genes, contained in the HIF-PPGL signature, that are upregulated in *VHL*-PPGL. GO/pathway enrichment.

Suppl Table S6.xlsx

**Table S7.** Association of HIF2$\alpha$ immunostaining and genetic/clinical data.

| | HIF2$\alpha$ immunostaining | | | | | |
|---|---|---|---|---|---|---|
| | PPGL | | | HNPGL | | |
| | positive | negative | *P* | positive | negative | *P* |
| *SDH/VHL* mutated | | | | | | |
| yes | 9 | 0 | 0.048 | 17 | 0 | 0.212 |
| no | 8 | 6 | | 13 | 2 | |
| Tumor location | | | | | | |
| extradrenal | 7 | 2 | 1 | | | |
| adrenal | 10 | 4 | | | | |
| carotid body | | | | 15 | 0 | 0.374 |
| vagal bulb | | | | 3 | 0 | |
| jugulo-tympanic bulb | | | | 11 | 2 | |
| parapharynx | | | | 1 | 0 | |
| Age at diagnosis | | | | | | |
| <45 years | 7 | 4 | 0.181 | 17 | 0 | 0.212 |
| >45 years | 8 | 1 | | 13 | 2 | |
| Gender | | | | | | |
| female | 5 | 4 | 0.132 | 22 | 2 | 0.556 |
| male | 12 | 2 | | 8 | 0 | |
| Metastatic disease | | | | | | |
| no | 6 | 1 | 0.382 | 29 | 2 | 0.938 |
| yes | 11 | 5 | | 1 | 0 | |

**Table S8.** Association of HIF1α and HIF2α expression.

| HIF2α | | HIF1α positive | HIF1α negative | *P* |
|---|---|---|---|---|
| PPGL (n = 21) | positive | 2 | 14 | 0.228 |
| | negative | 2 | 3 | |
| HNPGL (n = 30) | positive | 17 | 11 | 0.179 |
| | negative | 0 | 2 | |

**Table S9.** List of genes positively correlated with *EPAS1* in PPGL and GO/pathway enrichment.

Suppl Table S9.xlsx
