# Supplementary material for: Differential HIF2α Protein Expression in Human Carotid Body and Adrenal Medulla under Physiologic and Tumorigenic Conditions
Source: Cancers (Basel). 2022 Jun 17;14(12):2986. doi: 10.3390/cancers14122986 (PMC9221385; doi:10.3390/cancers14122986)

**Related to Figure 6**

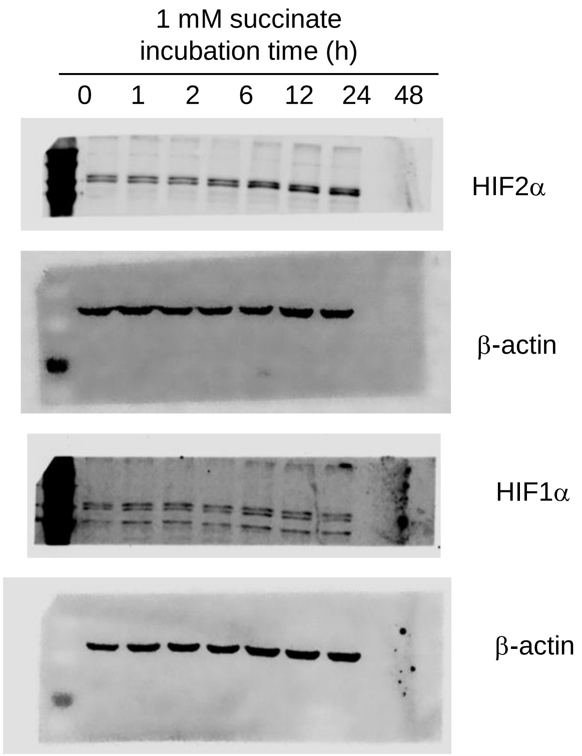


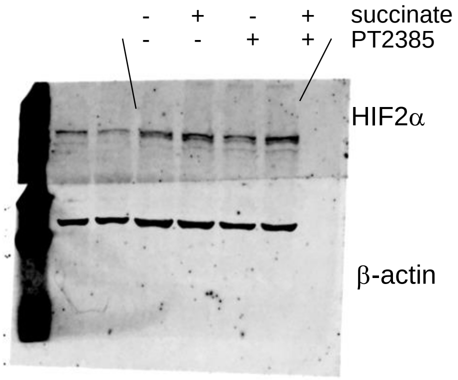


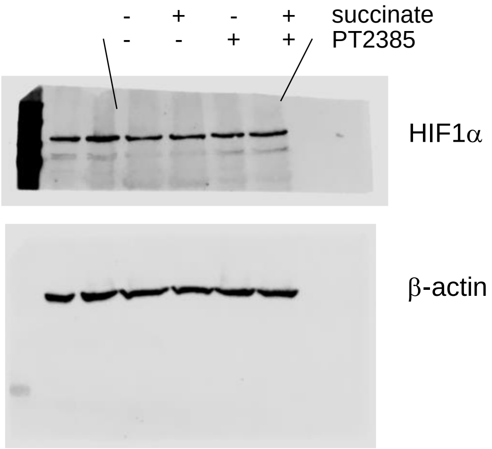


**Related to Supplementary Figure S1**

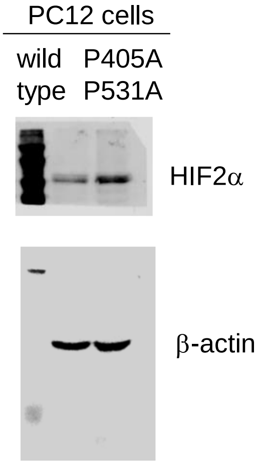


**Related to Supplementary Figure S3**

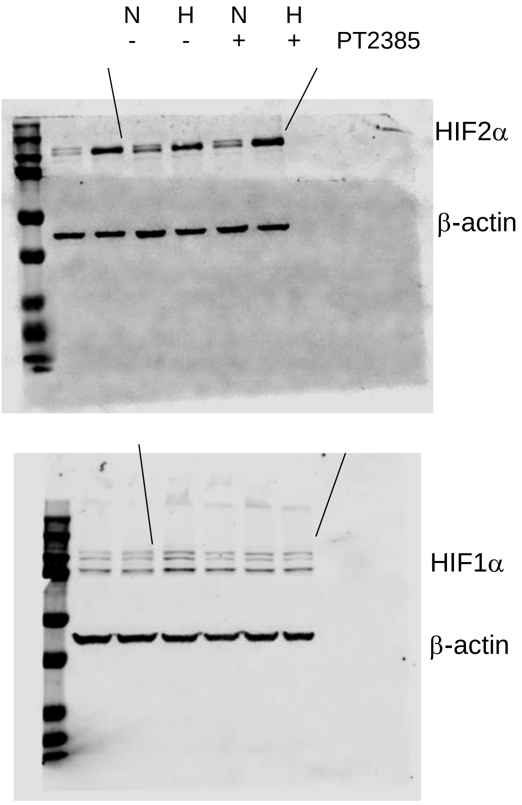


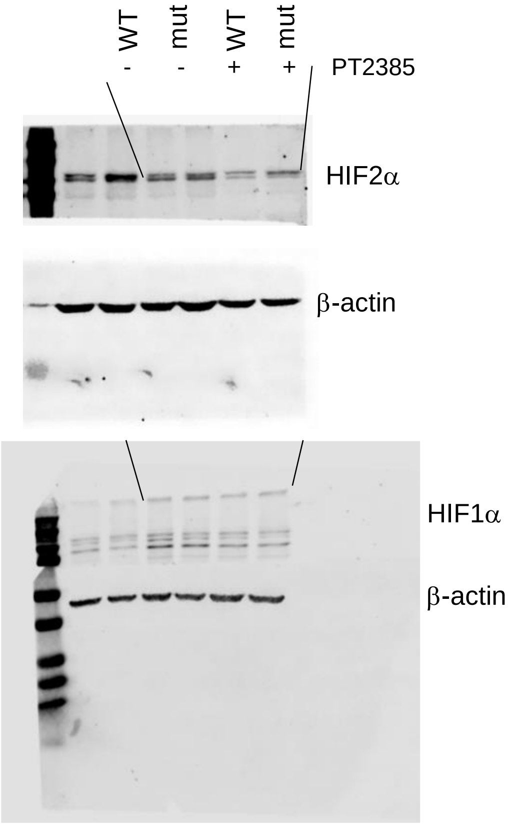

**Related to Supplementary Figure S4**

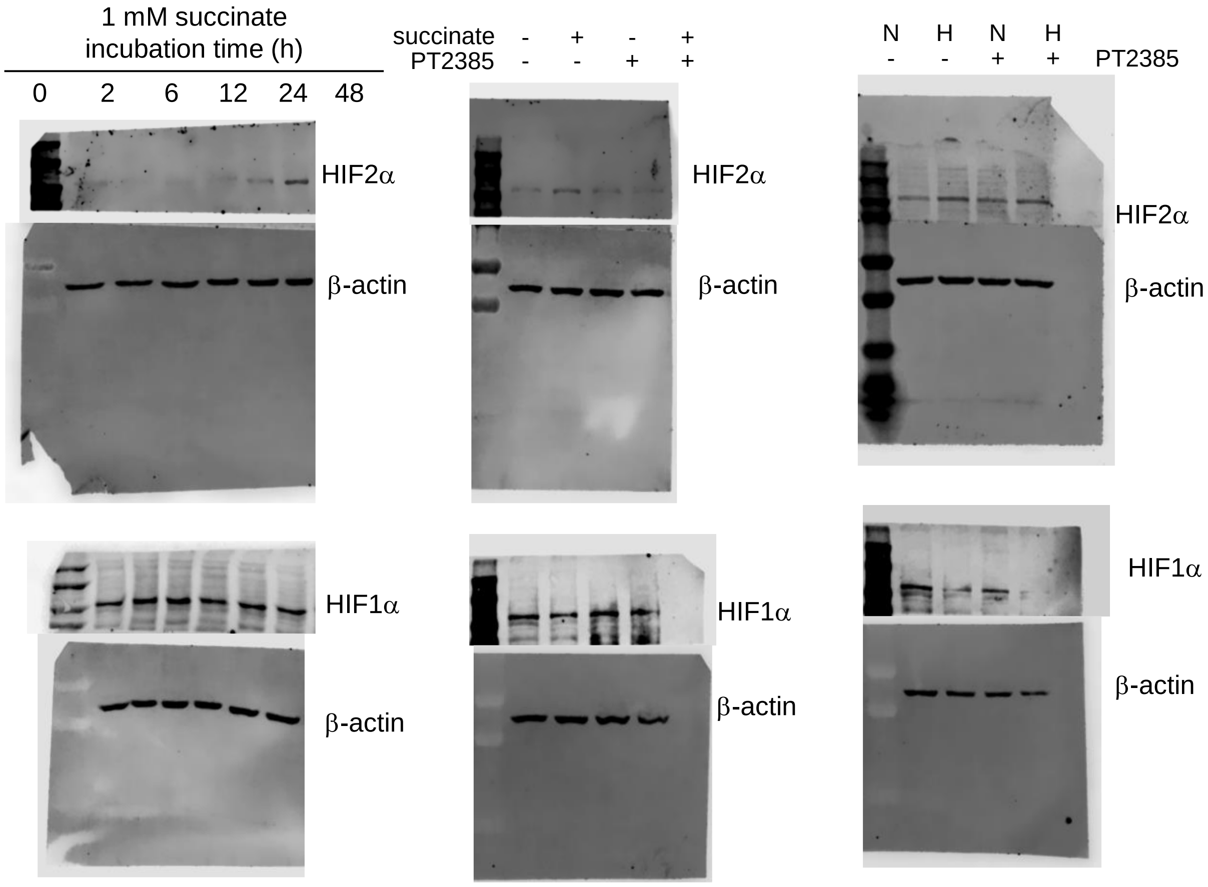

Supplement: Supplementary file 1 [file cancers-14-02986-s001.zip › Original images.pdf]
